# ERG transcription factors have a splicing regulatory function involving RBFOX2 that is altered in the EWS-FLI1 oncogenic fusion

**DOI:** 10.1093/nar/gkab305

**Published:** 2021-05-01

**Authors:** Olivier Saulnier, Katia Guedri-Idjouadiene, Marie-Ming Aynaud, Alina Chakraborty, Jonathan Bruyr, Joséphine Pineau, Tina O’Grady, Olivier Mirabeau, Sandrine Grossetête, Bartimée Galvan, Margaux Claes, Zahra Al Oula Hassoun, Benjamin Sadacca, Karine Laud, Sakina Zaïdi, Didier Surdez, Sylvain Baulande, Xavier Rambout, Franck Tirode, Martin Dutertre, Olivier Delattre, Franck Dequiedt

**Affiliations:** INSERM U830, Équipe Labellisée LNCC, PSL Research University, SIREDO Oncology Centre, Institut Curie, 75005 Paris, France; Université Paris Diderot, Sorbonne Paris Cité, F-75013 Paris, France; University of Liège, Interdisciplinary Cluster for Applied Genoproteomics (GIGA), Liège, Belgium; University of Liège, GIGA-Molecular Biology of Diseases, Liège, Belgium; INSERM U830, Équipe Labellisée LNCC, PSL Research University, SIREDO Oncology Centre, Institut Curie, 75005 Paris, France; Institut Curie, PSL Research University, CNRS UMR3348, INSERM U1278, F-91405 Orsay, France; Université Paris-Saclay, CNRS UMR3348, INSERM U1278, F-91405 Orsay, France; Équipe Labellisée Ligue Nationale Contre le Cancer, F-91405 Orsay, France; University of Liège, Interdisciplinary Cluster for Applied Genoproteomics (GIGA), Liège, Belgium; University of Liège, GIGA-Molecular Biology of Diseases, Liège, Belgium; INSERM U830, Équipe Labellisée LNCC, PSL Research University, SIREDO Oncology Centre, Institut Curie, 75005 Paris, France; University of Liège, Interdisciplinary Cluster for Applied Genoproteomics (GIGA), Liège, Belgium; University of Liège, GIGA-Molecular Biology of Diseases, Liège, Belgium; INSERM U830, Équipe Labellisée LNCC, PSL Research University, SIREDO Oncology Centre, Institut Curie, 75005 Paris, France; INSERM U830, Équipe Labellisée LNCC, PSL Research University, SIREDO Oncology Centre, Institut Curie, 75005 Paris, France; University of Liège, Interdisciplinary Cluster for Applied Genoproteomics (GIGA), Liège, Belgium; University of Liège, GIGA-Molecular Biology of Diseases, Liège, Belgium; University of Liège, Interdisciplinary Cluster for Applied Genoproteomics (GIGA), Liège, Belgium; University of Liège, GIGA-Molecular Biology of Diseases, Liège, Belgium; University of Liège, Interdisciplinary Cluster for Applied Genoproteomics (GIGA), Liège, Belgium; University of Liège, GIGA-Molecular Biology of Diseases, Liège, Belgium; INSERM U932, RT2Lab Team, Translational Research Department, PSL Research University, Institut Curie, F-75005 Paris, France; CNRS UMR5219, Institut de Mathématiques de Toulouse; Université de Toulouse; F-31062 Toulouse, France; INSERM U830, Équipe Labellisée LNCC, PSL Research University, SIREDO Oncology Centre, Institut Curie, 75005 Paris, France; INSERM U830, Équipe Labellisée LNCC, PSL Research University, SIREDO Oncology Centre, Institut Curie, 75005 Paris, France; INSERM U830, Équipe Labellisée LNCC, PSL Research University, SIREDO Oncology Centre, Institut Curie, 75005 Paris, France; Institut Curie, PSL Research University, NGS Platform, 26 rue d’Ulm, F-75005 Paris, France; University of Liège, Interdisciplinary Cluster for Applied Genoproteomics (GIGA), Liège, Belgium; University of Liège, GIGA-Molecular Biology of Diseases, Liège, Belgium; Claude Bernard University Lyon 1, INSERM 1052, CNRS 5286, Cancer Research Center of Lyon (CRCL), Lyon University, Lyon, France; Institut Curie, PSL Research University, CNRS UMR3348, INSERM U1278, F-91405 Orsay, France; Université Paris-Saclay, CNRS UMR3348, INSERM U1278, F-91405 Orsay, France; Équipe Labellisée Ligue Nationale Contre le Cancer, F-91405 Orsay, France; INSERM U830, Équipe Labellisée LNCC, PSL Research University, SIREDO Oncology Centre, Institut Curie, 75005 Paris, France; University of Liège, Interdisciplinary Cluster for Applied Genoproteomics (GIGA), Liège, Belgium; University of Liège, GIGA-Molecular Biology of Diseases, Liège, Belgium

## Abstract

ERG family proteins (ERG, FLI1 and FEV) are a subfamily of ETS transcription factors with key roles in physiology and development. In Ewing sarcoma, the oncogenic fusion protein EWS-FLI1 regulates both transcription and alternative splicing of pre-messenger RNAs. However, whether wild-type ERG family proteins might regulate splicing is unknown. Here, we show that wild-type ERG proteins associate with spliceosomal components, are found on nascent RNAs, and induce alternative splicing when recruited onto a reporter minigene. Transcriptomic analysis revealed that ERG and FLI1 regulate large numbers of alternative spliced exons (ASEs) enriched with RBFOX2 motifs and co-regulated by this splicing factor. ERG and FLI1 are associated with RBFOX2 via their conserved carboxy-terminal domain, which is present in EWS-FLI1. Accordingly, EWS-FLI1 is also associated with RBFOX2 and regulates ASEs enriched in RBFOX2 motifs. However, in contrast to wild-type ERG and FLI1, EWS-FLI1 often antagonizes RBFOX2 effects on exon inclusion. In particular, EWS-FLI1 reduces RBFOX2 binding to the *ADD3* pre-mRNA, thus increasing its long isoform, which represses the mesenchymal phenotype of Ewing sarcoma cells. Our findings reveal a RBFOX2-mediated splicing regulatory function of wild-type ERG family proteins, that is altered in EWS-FLI1 and contributes to the Ewing sarcoma cell phenotype.

## INTRODUCTION

ERG (E-26 transformation specific-related gene) family proteins (ERG, FLI1 and FEV) belong to the larger family of ETS transcription factors (TFs), that is one of the largest families of TFs in metazoans and is defined by a highly conserved DNA-binding ETS domain (1). According to the current model, ERG family proteins act as canonical TFs, binding to specific DNA sequences in promoters and enhancers through their ETS domain and regulating expression of their target genes (2). Our recent findings have led us to challenge this view. Indeed, we reported that in addition to their role in transcription, ERG family proteins also impact gene expression through regulation of mRNA decay (3). However, whether ERG proteins may be involved in additional steps of the mRNA life is unknown.

*ERG* family genes are implicated in oncogenic gene fusions due to translocations that typify several cancers. These include prostate cancers (4), myeloid leukemias (5) and Ewing sarcoma (6), a highly aggressive bone and soft tissue tumor. Because they consistently include the C-terminal half of the ERG protein, which contains the ETS DNA-binding domain, ERG fusions have mostly been studied as oncogenic TFs. Indeed, these fusions acquire specific transcriptional properties that are not shared by wild-type (wt) ERG factors. For instance, EWS-FLI1, the primary oncogenic fusion of Ewing sarcoma gains the ability to bind and epigenetically convert silenced GGAA microsatellites into active enhancers (7,8). In addition to its transcriptional activity, EWS-FLI1 has been shown to influence alternative splicing of pre-messenger RNAs (pre-mRNAs) through interactions with core components of the spliceosome or through regulation of RNA polymerase II elongation rate (9–12). Because of the functions of EWS in various stages of mRNA metabolism, including splicing (13,14), the splicing activity of EWS-FLI1 has been attributed to its EWS moiety. Indeed, wild-type ERG family proteins have not been shown to be involved in splicing regulation.

Beyond regulating pre-mRNA synthesis, TFs can also affect downstream steps of gene expression. In particular, a number of studies have reported cases of TFs involved in pre-mRNA splicing regulation (15). However, these studies almost exclusively describe indirect mechanisms, in which TFs impact pre-mRNA splicing by modification of RNA polymerase II elongation rate, recruitment of transcriptional coactivators that affect splicing, or modulation of the expression of direct splicing regulators (e.g. core spliceosome components and other splicing factors) (16). More recently, it was shown that some TFs bind directly to pre-mRNA and control alternative splicing via unknown yet direct mechanisms (17).

In this study, we identify and characterize a novel non-transcriptional function of wild-type ERG family proteins in alternative splicing of pre-mRNAs. While wild-type ERG and FLI1 proteins cooperate with the splicing regulator RBFOX2, EWS-FLI1 represses a subset of the RBFOX2-dependent mesenchymal splicing program, including an isoform of the Adducin 3 (*ADD3*) gene involved in cytoskeleton remodeling (18).

## MATERIALS AND METHODS

### Plasmids

Constructs encoding human FLI1, ERG and FEV have been described elsewhere (3). Open reading frames (ORF) encoding human RBFOX1, RBFOX2 and EWS were obtained as pDONR223 from the human ORFeome v5.1 (Center of Cancer Systems Biology (CCSB), Dana-Farber Cancer Institute (DFCI)). Isolated domains and deletion variants of ERG, and EWS and ERG fusions were inserted into pDONR223 by Gateway cloning (Invitrogen) using specific primers flanked in 5′ by the following AttB1 and AttB2 Gateway sites (Forward: 5′-GGGGACAACTTTGTACAAAAAAGTTGGC(ATG)-3′(AttB1); Reverse: 5′-GGGGACAACTTTGTACAAGAAAGTTGA-3′ (AttB2)). Inserts were transferred from the pDONR223 into destination vectors (N-terminal tags): pDEST1899 (FLAG), pDEST1899-MS2-CP (FLAG-MS2-CP Nter tag), pDEST475 (HA) (kind gifts of James L. Hartley and Dominic Esposito, SAIC-Frederick Inc.), and Gateway modified pGEX-2TK (GST) (kind gift from Pascal Braun, CCSB, Dana-Farber Cancer Institute). pDEST1899-FLAG Erg ORFs were subcloned in the pN-MS2-CP (MS2 Nt) described in (19), following classical cloning procedures. All constructs were verified by sequencing.

### Cell lines

Cell lines were obtained from American Type Culture Collection (ATCC) and were routinely checked for mycoplasma. Human Umbilical Vein Endothelial Cells (HUVECs) were obtained from Lonza. Ewing sarcoma cell line A673/TR/shEF (also called ASP14) was generated as previously described (20) and MHH-ES1 has been ordered from the DSMZ collection. All cell lines, except for HL-60 and HUVECs were cultured at 37°C, in 5% CO_2_ with DMEM (Gibco) supplemented with 10% FBS (Eurobio) and 1% antibiotics (penicillin and streptomycin, P/S (Gibco)). HL-60 were cultured in RPMI (Gibco), 10% FCS and 1% P/S. HUVECs were grown at 37°C, in endothelial growth medium (EGM-2, Lonza, UK) and passages 2–7 were used for the study.

Induction of EWS-FLI1 specific shRNA was performed by adding 1 μg/ml of doxycycline (DOX) in the medium *ex-tempo*. For reversion studies, doxycycline was removed after 7 days and cells were washed three times to stop the shRNA induction.

### DNA and siRNA transfection

Plasmids were transfected in HeLa cells with the Lipofectamine 2000 Transfection System according to the manufacturers’ protocol (Thermo Fisher Scientific) and in HEK293 cells using the phosphate-calcium method, unless otherwise stated. Cells were collected 36–48 h post-transfection.

Transfection of siRNAs (see sequences in Supplementary Table S13) was performed using Lipofectamine RNAiMAX Reagent (Thermo Fisher Scientific) or JetPRIME (Polyplus) according to the manufacturer's instructions. SiRNA transfections in HUVECs were performed using the GeneTrans 2 (MoBiTec) reagents according to the manufacturer's protocol. Cells were collected 48 or 72 h post-transfection.

### Western blot

Cells were washed once with cold PBS and scraped on ice with lysis buffer (20 mM Tris–HCl pH 8; 1% NP40; 150 mM NaCl) supplemented with protease inhibitor cocktail (Sigma). Protein lysates were quantified using Bradford protein assay (BioRad). Alternatively, total cell extracts were prepared by lysing cells directly into Laemmli buffer. Proteins extracts were separated by sodium dodecyl sulfate-polyacrylamide gel (SDS-PAGE) electrophoresis and transferred onto nitrocellulose membrane. Membranes were incubated with primary antibodies followed by horseradish peroxidase-conjugated anti-IgG. Proteins were detected using enhanced chemiluminescence (Pierce) and images were acquired with ChemiDoc™ Gel Imaging System (Bio-Rad). Quantifications were performed using the ImageJ software. Primary antibodies are listed in Supplementary Table S13.

### MS2-based tethering assay

MS2-based tethering assays were performed using HeLa cells co-transfected with control MS2-CP or various MS2-CP-tagged constructs, together with SMN2-MS2 minigene (21). Reverse transcription (RT)-PCR amplifications were performed (see below) using forward and reverse primers of SMN2-MS2 minigene (See Supplementary Table S13).

### Protein complementation assay

ORFs corresponding to RBFOX2 and ERG full-length or ERG lacking the CTAD region were cloned in pSPICA-N1 or pSPICA-N2 destination vectors containing the GLucN1 and GLucN2 fragments of the Gaussia princeps luciferase, respectively (22). HEK293T cells were cultured in 24-well plates and transfected with 500 ng of the appropriate constructs (GLucN1 + GLucN2) using polyethylenimine (PEI). The DNA/PEI ratio (mass:mass) was 1:3. Cell medium was removed 24 h post-transfection. Two hundreds μL of the Renilla Luciferase Assay Lysis Buffer (Renilla Luciferase Assay System, Promega) were added in each well and cell lysis was performed under vigorous shaking for 20 min. Thirty microliters of cell lysates were plated in 96-well White Flat Bottom plates in triplicates. Luminescence was measured by auto-injecting 30 μl per well of Renilla Luciferase substrate using a Centro XS3 LB 960 luminometer (Berthold Technologies; 1 sec delay time; 10 s integration time).

### Subcellular fractionation

HeLa (treated or not for 2 h with 5 μM Actinomycin D) or HL-60 cells were washed with cold PBS, harvested and lysed with CLB buffer [Cytoplasmic Lysis Buffer; 10 mM Tris–HCl pH 7.9, 340 mM Sucrose, 3 mM CaCl_2_, 0.1 mM EDTA, 2 mM MgCl_2_ 1 mM DTT, 0.5% NP40, cOmplete Protease Inhibitor Cocktail (Roche) and Halt Phosphatase Inhibitor Cocktail (Thermo Fisher Scientific)] on ice for 5 min. The cytoplasmic fraction was removed by centrifugation at 3500 × g for 15 min at 4°C. The pellet was washed several times with CLB wash buffer (CLB buffer without NP40) and lysed with NLB buffer [Nuclear Lysis Buffer; 20 mM HEPES pH 7.9, 10% glycerol, 3 mM EDTA, 150 mM KOAc, 1.5 mM MgCl_2_, 1 mM DTT, 0.1% NP40, cOmplete Protease Inhibitor Cocktail (Roche), Halt Phosphatase Inhibitor Cocktail and Protector RNase Inhibitor (Roche)]. Soluble and Chromatin/HMW fractions were separated by centrifugation at 15 000 × g for 30 min at 4°C. The pellet was washed several times with NLB washing buffer (NLB buffer without NP40), centrifuged at 3500 × g for 5 min and lysed with NIB buffer (Nuclease Incubation Buffer; 150 mM HEPES pH 7.9, 10% Glycerol, 150 mM KOAc, 1.5 mM MgCl_2_, 1 mM DTT, cOmplete Protease Inhibitor Cocktail and Halt Phosphatase Inhibitor Cocktail). Next, the lysate was divided into two equal portions, one of which was treated with 10 μg/ml of RNase A (Thermo Fisher Scientific) for 30 min at RT. Then, lysates were centrifuged at 20 000 × g for 30 min and supernatants were recovered as the RNA-associated chromatin fraction. The pellets were incubated at 25°C on a rotator with 5 U/μl of Benzonase (Sigma) to solubilize the DNA-associated fraction. Cell fractions were then analyzed by SDS-PAGE.

### Immunoprecipitation

For endogenous co-immunoprecipitation, HeLa cells were lysed in IPLS buffer [ImmunoPrecipitation Law Salt; 50 mM Tris–HCl pH 7.5, 0.5 mM EDTA pH 8, 0.5% NP-40, 10% glycerol, 120 mM NaCl, cOmplet Protease Inhibitors (Roche) and Halt Phosphatase Inhibitors (Thermo Scientific)]. Lysates were incubated with Protein G magnetic beads (Millipore) for 1 h at 4°C for preclearing and incubated at 37°C for 30 min with or without RNase A (200 μg/ml) (Thermo Scientific). Then, lysates were incubated for 2 h at 4°C with Protein G magnetic beads (Millipore) and anti-ERG antibody or rabbit anti-IgG antibody (Santa Cruz). Beads were washed 4 times with IPLS buffer. Immunoprecipitates were boiled in Laemmli buffer and analyzed by SDS-PAGE and Western blot according to standard procedures and developed with the ECL detection kit (GE Healthcare Bio-Sciences, Uppsala, Sweden).

For co-immunoprecipitation of overexpressed proteins, HEK293 cells overexpressing ERG-FLAG or deletion variants of ERG and HA/MYC-tagged proteins were lysed in IPLS buffer. Lysates were then incubated with anti-FLAG M2 magnetic beads (Sigma-Aldrich) for 2 h at 4°C, washed 3 times with IPLS lysis buffer, twice with IPMS buffer (ImmunoPrecipitation Medium Salt: IPLS with 500 mM NaCl) and twice with IPLS buffer. Immunoprecipitates were either boiled in Laemmli buffer and analyzed by SDS-PAGE or selectively eluted from anti-FLAG M2 magnetic beads by 3 successive elutions using 3XFLAG peptide (0.2 mg/ml in IPLS buffer, 3 times 20 min at 4°C) (ApexBio). Eluates were boiled in Laemmli and analyzed by SDS-PAGE and western blot according to described procedures.

### RT-PCR/qPCR

Total RNA was isolated using the Nucleospin II kit (Macherey-Nagel) and reverse-transcribed using the High-Capacity cDNA Reverse Transcription kit (Applied Biosystems). Next, cDNA molecules were amplified by PCR using the AmpliTaqGold DNA Polymerase kit with Gold Buffer. One μg of template total RNA was used for each reaction. The resulting cDNA was diluted between 10- and 100-fold, depending on the abundance of targets. Between 1 and 3 μl of diluted cDNA were used for PCR/qPCR amplifications. Oligonucleotides were purchased from MWG Eurofins Genomics or Eurogentec (Supplementary Table S13). PCR reactions were loaded on 2% agarose gel electrophoresis with SYBR Safe DNA Gel Stain (1/10 000, Invitrogen). Gels were observed and photographed on a UV lamp and images were analyzed by densitometry using the ImageJ software.

All Quantitative PCR reactions were performed on ABI/PRISM 7500 instrument and analyzed with 7500 system SDS software. The amplification cycle was composed of an incubation at 50°C for 2 min, followed by an initial denaturation step at 95°C for 10 min, 45 cycles at 95°C for 10 s and 60°C for 45 s. HPLC-purified oligonucleotides were purchased from MWG Eurofins Genomics or Eurogentec. Primer specificities were evaluated *in sili*co using a blast homology search and assessed post-amplification by examination of the melt curve. Primer efficiencies were evaluated by the PCR standard curve method and only primers with >98% efficiency were used. Experiments were carried out in triplicate for each data point and final results are presented as average of at least three biological replicates. Relative quantification of targets, normalized to an endogenous control, was performed using the comparative 2^–ΔΔCt^ method. Using this method, we obtained the fold changes in gene expression or enrichment, normalized to an internal control (for gene expression levels) or to input (for RIP experiments). Error bars indicate SD. Primers used are listed in Supplementary Table S13.

### RNA-immunoprecipitation-qPCR analysis

Dynabeads Protein G (ThermoFisher Scientific) were incubated with anti-RBFOX2 antibody (A300-864A, Bethyl Laboratories), anti-ERG antibody (ab133264, Abcam) or normal rabbit IgG (Santa Cruz) at 4°C overnight with rotation. Before cell harvesting, RNA–protein complexes were crosslinked with 1% formaldehyde (incubation at RT for 10 min) and crosslink reaction was quenched using 125 mM glycine for 5 min at RT. Cells were washed twice with ice-cold PBS before harvesting and pelleted by centrifugation (4°C, 5 min, 800 × g). Cells were resuspended in 1 ml RIPA (Sigma) with Complete Protease Inhibitor Cocktail (Sigma) at 1× concentration and 1 μl RNAse inhibitor (RNaseOUT, Thermo Fisher Scientific). Cell lysates were sonicated for 5 min. Supernatant was collected after centrifugation at 4°C for 10 min (10 000 × g). An aliquot was used for RNA input and was treated with proteinase K before RNA extraction with TRIzol Reagent (Thermo Fisher Scientific). For immunoprecipitation, 400 μg of protein were incubated with antibody-loaded Dynabeads overnight with rotation at 4°C. Supernatant was removed and beads were washed twice with RIPA buffer before RNA-protein complex elution by incubation with elution buffer (Tris–HCl pH 8 100 mM; Na2-EDTA 10 mM; 1% SDS in H_2_O) 3 min at 90°C. Proteins were digested with proteinase K treatment and RNA was extracted with TRIzol Reagent (ThermoFisher Scientific) for RT-qPCR analysis.

### RNA sequencing and data processing

EWS-FLI1-dependent splicing events were identified by comparing doxycycline-untreated versus doxycycline-treated A673/TR/shEF cells. ERG-, FLI1 or RBFOX2-dependent splicing events were identified by comparing cells treated with siCTRL or with the corresponding siRNA. RNA was isolated as described above and sample integrity was evaluated using a Bioanlayzer instrument (Agilent). Only samples with RNA Integrity Number above 9 were used. Libraries were performed using the TruSeq Stranded mRNA Library Preparation Kit. Equimolar pools of libraries were sequenced on a Illumina HiSeq 2500 machine using paired-end reads and High Output run mode allowing 200 million raw reads per sample. Raw reads were mapped to the human reference genome hg19 using the STAR aligner (v.2.5.0a) (23). PCR-duplicated reads and low mapping quality reads (MQ<20) were removed using Picard tools and SAMtools, respectively. We next used rMATS (v3.0.9) (24), an event-based tool, to identify differentially spliced events using RNA-seq data. Five distinct alternative splicing events were analyzed using rMATS: skipped exons (SE), alternative 3′ splice sites (A3SS), alternative 5′ splice sites (A5SS), mutually exclusive exons (MXE) and retained introns (RI). Briefly, rMATS uses a count-based model, to calculate percent of spliced-in (PSI) value among replicates, using both spliced reads and reads that mapped to the exon body. We used three different thresholds to identify differentially spliced events between two groups: each splicing event has to be (i) supported by at least 15 unique reads, (ii) |ΔPSI| > 10%; (iii) FDR < 0.05. For RT-qPCR validation, we choose a set of alternative splicing events with ΔPSI values spread across a wide range. Events were selected from a list of events with >50 reads supporting the event in at least one condition (to allow detection by RT-PCR).

Gene expression analysis was performed as follows: aligned reads were counted using htseq-count v.0.6.1p1 (25) and normalized according to the DESeq size factors method (26). We used fold change ≥2 and FDR <0.05 as the determination of differentially expressed genes (DEG).

Statistical analysis and plots were performed inside R environment version 3.1.0. Fastq files have been deposited to the NCBI repository under the accession number PRJNA521683.

### Motif enrichment analysis

To identify the RNA-binding proteins (RBPs) around skipped exons, we extended the rMAPS software (27). This tool identifies the binding positions of RBPs around skipped exons. The purpose of rMAPS is to identify known RBP motifs that are significantly enriched in differentially regulated exons between two sample groups as compared to control (background) events. rMAPS analyzes each set of 300 nt length sequences, with a sliding window of 50 nt, and counts the number of times the motif matches each sequence. The resulting ‘enrichment score’ is then used to compare local enrichment in the window between significant exons and background exons by the Wilcoxon rank sum test. This process results in a set of 250 highly correlated *P*-values, which rMAPS summarizes by the minimum (raw) *P*-value.

We extended rMAPS by proposing a method to identify intervals significantly enriched for a given RBP. Here, ‘significantly’ means that with high probability, the proportion of false positives (or False Discovery Proportion: FDP) among any of the selected intervals does not exceed a user-defined threshold. This method is based on the concept of post-hoc inference, as introduced by Goeman and Solari (28) and further studied by Blanchard, Neuvial and Roquain (29). Importantly, the user may choose the threshold on the FDP post hoc, i.e. after data analysis. Compared with rMAPS, this approach reduces the number of identified false positives and allows the identification of their precise binding site. We chose to call significant all the intervals with a post-hoc false discovery proportion (ph-FDP) <25%. This value represents the maximum frequency of false positives present in each set of *P*-values called significant.

### ChIP-Seq analysis

To study the binding of ERG and FLI1 to DNA in HUVEC cells, we used ChIP-Seq data available on GEO: GSE109696 for ERG, FLI1 and H3K4me3 binding (30) and GSE124891 for the control. Reads were aligned to the human reference genome (GRCh37/hg19) with bowtie2 2.2.9 (31). Uninformative reads (multimapped reads, duplicated reads and reads with low mapping score) were filtered out with samtools 1.3 (32). Peaks were called with MACS2 2.1.1 (33) with the narrow option and input DNA as control. Bedtools v2.21.0 (34) was used to compare peaks at transcription start sites (TSS) of ERG- or FLI1-dependent alternatively spliced genes or 250bp around ERG- or FLI1-regulated ASEs. For TSS, we considered all ERG and FLI1 peaks overlapping with TSS. For exons, we removed ERG and FLI1 peaks overlapping with H3K4me3 peaks and evaluated the presence of peaks within a window spanning from 250 bp upstream to 250 pb downstream of the alternative exon.

### Patient data

RNA-sequencing datasets from Ewing sarcoma patients were previously published (35) and are available at EGA under the accession number EGAS00001003333. Clinical data are available at the ICGC data portal (project reference: BOCA-FR).

## RESULTS

### A role for ERG in the control of pre-mRNA splicing

**Figure 1.**
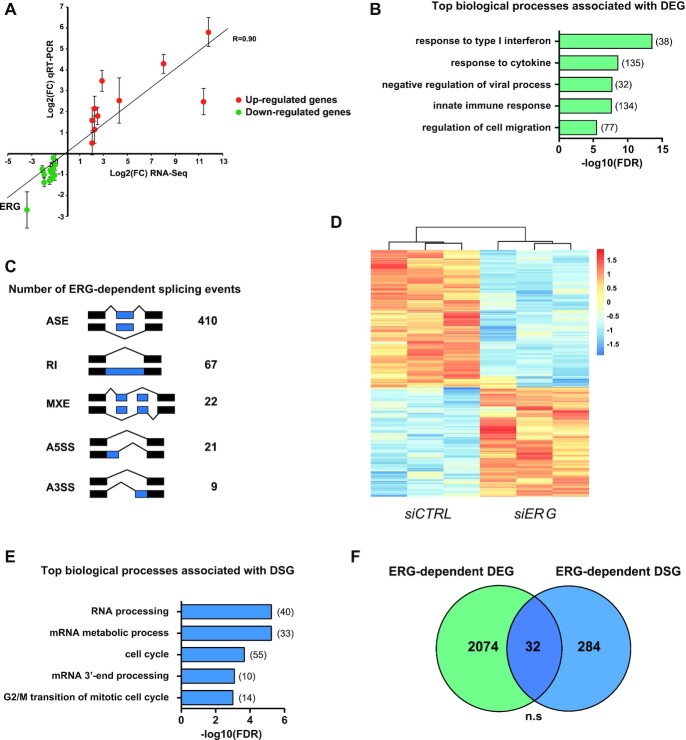
ERG controls the HeLa transcriptome at the levels of gene expression and alternative splicing. (**A**) Correlation between mRNA levels quantified by RNA-sequencing or qRT-PCR on a set of 22 target mRNAs expected to be up- (FDR<0.05, Log2(FC)>1, red dots) or down- (FDR < 0.05, log_2_(FC) < 1, green dots) regulated after ERG knockdown in HeLa cells. Results shown are means ± SD (*n* = 3 independent experiments). (**B**) Distribution of enriched GO biological process terms in differentially expressed genes (DEG) following ERG knockdown in HeLa cells. (**C**) Numbers of significantly differentially spliced events identified by rMATS after ERG knockdown in HeLa cells with the following criteria: supported by at least 15 unique reads, |ΔPSI| > 10% and FDR < 0.05. ASE: Alternatively spliced exons, RI: Retained intron, MXE: Mutually exclusive exons, A5SS: Alternative 5′ splice site, A3SS: Alternative 3′ splice site. (**D**) Heatmap of *Z*-scores of percent of spliced-in (PSI) values from significantly differentially spliced exons between HeLa cells transfected either with *siCTRL* or *siERG*. (**E**) Distribution of enriched GO biological process terms associated with differentially spliced genes (DSG, i.e. genes with at least one ERG-regulated ASE) following ERG knockdown in Hela cells. (**F**) Overlap between differentially expressed genes and differentially spliced genes (DSG) in HeLa cells after ERG knockdown. Expected overlap = 39. n.s.: not significant.

Our recent observation that ERG TFs control gene expression through regulation of mRNA stability prompted us to investigate whether they might also be involved in other co- or post-transcriptional steps of mRNA processing, including splicing (3). Toward this aim, we down-regulated *ERG* expression in HeLa cells and analyzed transcriptomic changes by RNA-seq. We chose HeLa cells because they predominantly express ERG and very little of FLI1 and FEV, the other two ERG family members (Supplementary Figure S1A). Transfection with siRNA led to a reduction of ERG protein levels to <10% of its normal levels (Supplementary Figure S1B). Differential analysis of mRNA expression levels between control and ERG-depleted cells identified 2106 genes whose expression level was significantly altered by at least 2-fold following *ERG* knockdown, including 945 (45%) up- and 1160 (55%) down-regulated (Supplementary Table S1). Changes in gene expression were confirmed by RT-qPCR on selected genes, thus validating our differential expression analysis pipeline (Figure 1A). Differentially expressed genes (DEG) showed significant enrichment for Gene Ontology (GO) biological process terms associated with interferon-gamma response and regulation of cell migration (Figure 1B). Splicing analysis revealed that ERG is significantly associated with a large number of alternative splicing alterations (Figure 1C). By far, the most frequent splicing event (77.5%, 410/529) was regulation of alternatively spliced exons (ASEs, also called cassette exons). These 410 ASEs were consistent across three biological replicates and included 228 spliced-in (i.e. alternative exons preferentially skipped after *ERG* knockdown) and 182 spliced-out (i.e. alternative exons preferentially included after *ERG* knockdown) exons in control cells as compared to ERG-depleted cells (Figure 1D; Supplementary Table S2). GO analysis revealed that differentially spliced genes (DSG) were significantly associated with various biological processes related to mRNA metabolism and cell cycle progression (Figure 1E). Interestingly, we found no significant overlap between differentially spliced and differentially expressed genes, indicating that ERG controls independent sets of genes at the transcription and splicing levels (Figure 1F).

**Figure 2.**
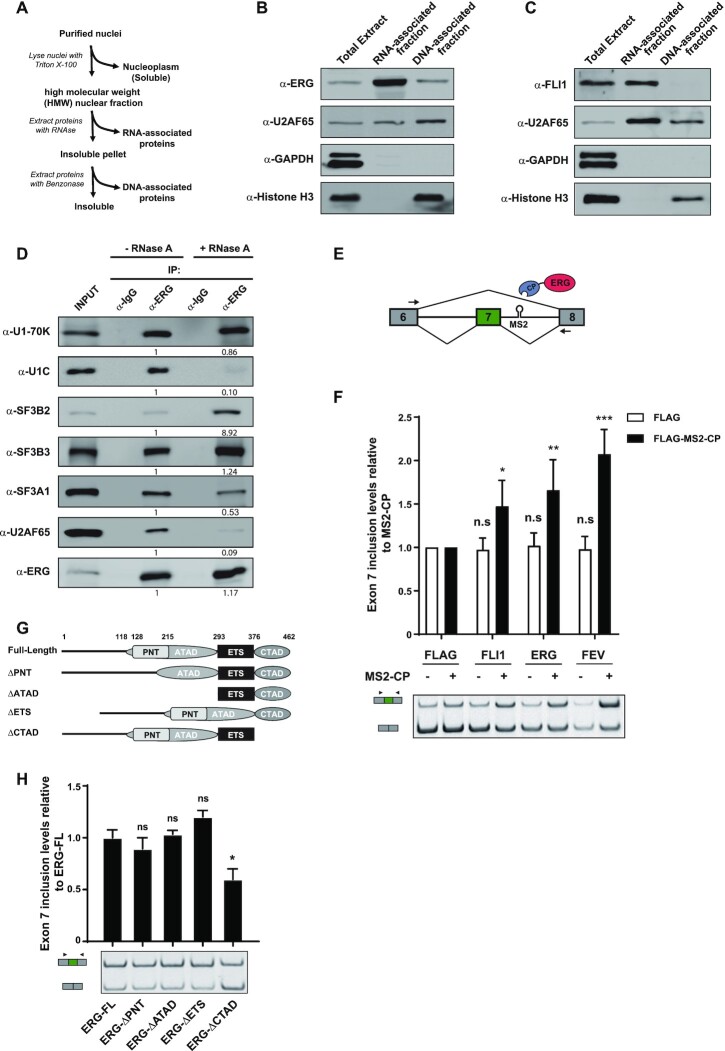
ERG associates with core components of the splicing machinery to control mRNA splicing. (**A**) Preparation of the nuclear HMW, RNA- and DNA-associated fractions from HeLa cells. (**B**) Immunoblot analysis of ERG in total, RNA- and DNA-associated HMW fractions from HeLa cells. U2AF65, GAPDH and Histone H3 specific antibodies were used as control for fraction purity. (**C**) Immunoblot analysis of FLI1 in total, RNA- and DNA-associated HMW fractions from HL-60 cells. U2AF65, GAPDH and Histone H3 specific antibodies were used as control for fraction purity. (**D**) Western blot analysis of endogenous ERG immunoprecipitates from RNAse A-treated or untreated lysates from HeLa cells, using antibodies against the indicated spliceosome components (SF3B2, SF3B3, SF3A1, U170-K, U1C, U2AF65). An anti-ERG antibody was used to control the immunoprecipitation efficiency. Quantification of the protein levels was performed by signal densitometry relatively to the α-ERG immunoprecipitation from untreated lysate. (**E**) Diagrams of the *SMN2* minigene reporter and MS2-fused ERG constructs. An MS2 binding site is inserted in the intron downstream from exon 7. The primers for RT–PCR are indicated by arrows. (**F**) RT-PCR analysis of *SMN2* minigene exon 7 inclusion. Samples are RNA from HeLa cells transfected with the *SMN2-MS2* minigene reporter and either FLAG- or FLAG-MS2-CP-tagged versions of FLI1, ERG and FEV. Results shown are means ± s.e.m (n = 3 independent experiments) relative to FLAG-MS2-CP alone. *P < 0.05; **P < 0.01; ***P < 0.001; n.s.: non-significant by two-tailed unpaired Student's *t*-test. (**G**) Representation of ERG-deletion constructs. Numbers indicate positions of amino-acids. (**H**) RT-PCR analysis of *SMN2* minigene exon 7 inclusion. Samples are RNA from HeLa cells transfected with the *SMN2-MS2* minigene reporter and FLAG-MS2-CP-tagged version of the indicated ERG deletion variants. Results shown are means ± s.e.m. (*n* = 3 independent experiments) relative to ERG full-length protein (ERG-FL). *P<0.05; n.s: non-significant by two-tailed unpaired Student's *t*-test.

These observations raised the intriguing possibility that ERG might have a direct role in splicing regulation, independent of its transcriptional effects. If ERG can act as a direct splicing regulator, we expected it to be found in association with nascent RNA, as splicing mostly occurs co-transcriptionally (36). To test this, we prepared a fraction corresponding to chromatin and other high molecular weight nuclear components (HMW) from nuclei of HeLa cells. We then extracted RNA- and DNA-associated proteins from the insoluble Chromatin/HMW fraction by treating the pellet sequentially with RNase A and benzonase, according to a previously described protocol (37) (Figure 2A). ERG was efficiently extracted from the Chromatin/HMW fraction by RNase A treatment, indicating that a portion of ERG associates with chromatin in a RNA-dependent manner (Figure 2B). Inhibiting transcription using actinomycin D reduced the presence of ERG in the RNA-associated fraction, suggesting that association of ERG with chromatin is mediated by nascent RNA (Supplementary Figure S2A). As expected, ERG was also found in the DNA-associated chromatin fraction, as it was solubilized with an additional benzonase treatment of the RNase-insensitive pellet (Figure 2B). Consistent with these findings, FLI1, another member of the ERG family, was also present in both the RNA- and DNA-associated fractions of HL-60 cells expressing FLI1 endogenously (Figure 2C).

Curation of two protein-protein interaction databases (38,39) (BioGRID, https://thebiogrid.org and STRING, https://string-db.org) identified 97 unique interactors for ERG (Supplementary Table S3). Enrichment analysis for GO biological processes terms revealed that a significant proportion of ERG binding partners (27%; 26 out of 97; FDR = 7.77E–22) were categorized as ‘mRNA splicing, via spliceosome’ (GO:0000398) (Supplementary Figure S2B). These proteins include core components of small nuclear ribonucleoprotein (snRNP) particles as well as snRNP-associated factors, spliceosome-associated hnRNPs and pre-mRNA processing factors. By immunoblot analysis of ERG immunoprecipitates, we confirmed the association of endogenous ERG with core components of the spliceosome. These included two U1 snRNP proteins, U1–70K and U1C; U2AF65, the larger subunit of U2 Small Nuclear RNA Auxiliary Factor; and U2 snRNP-associated proteins SF3B2, SF3B3 and SF3A1 (Figure 2D). Most of these interactions were maintained when lysates were treated with RNase A. In contrast, a significant reduction of the amounts of U1C and U2AF65 co-immunoprecipitating with ERG was observed, indicating that the association of ERG with these factors is mediated by RNA. Altogether, these data indicate that a fraction of ERG is engaged in a complex network of interactions with spliceosomal proteins and is present on chromatin-bound nascent RNA, consistent with a possible direct role in pre-mRNA splicing.

To formally test this possibility, we used a splicing reporter assay in which an MS2-binding site was inserted downstream of exon 7 in a minigene that contains exons 6 to 8 of the *SMN2* gene (21). FLAG-tagged ERG members were expressed with an N-terminal tag derived from the MS2 bacteriophage coat protein (CP) to enable their direct tethering to the minigene transcript (Figure 2E). Compared to MS2-CP alone, tethering of ERG, FLI1 or FEV onto the intronic MS2 site significantly increased inclusion of exon 7 (Figure 2F, black bars). Importantly, compared to FLAG alone, ERG family proteins had no effect when not fused to MS2-CP (Figure 2F, white bars), indicating that their ability to control exon inclusion of the reporter minigene strictly relies on their recruitment to the target pre-mRNA. To identify the domain of ERG responsible for this effect, we generated a series of MS2-ERG fusion constructs lacking the PNT (pointed), ATAD (amino-terminal activation domain), ETS (DNA-binding) or CTAD (carboxy-terminal activation domain) domains, and tested their effects on the *SMN2* reporter (Figure 2G). Among these constructs, the variant lacking the CTAD (ERG-ΔCTAD) was the only one showing a significantly reduced effect on exon inclusion (Figure 2H). These results indicate that the CTAD, which is shared among the ERG subfamily members but not other ETS family members (Supplementary Figure S2C), is important for their ability to promote exon inclusion. Because the variant lacking the ETS domain is unable to bind DNA (40) but still promotes inclusion of the reporter exon, these results are consistent with the idea that the function of ERG in pre-mRNA splicing is direct, requiring its recruitment to pre-mRNA and independent of its transcriptional activity.

### ERG and RBFOX2 control a common splicing program in HeLa cells

**Figure 3.**
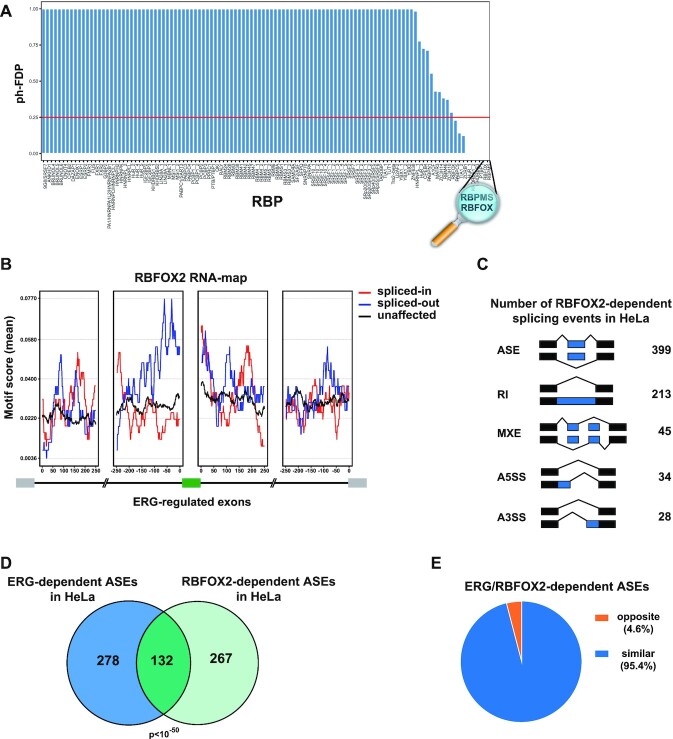
ERG regulates an RBFOX2-dependent splicing program. (**A**) Post-hoc false discovery proportion (ph-FDP) values of motif enrichment analysis performed on ERG-regulated spliced-out exons in HeLa cells. Red line represents the upper-bound of the significant RBPs at 25% FDP. (**B**) RBFOX motif enrichment analysis upstream and downstream of ERG-regulated exons in HeLa cells. Red and blue lines represent intronic RBFOX motif scores around ERG spliced-in or spliced-out exons, respectively. Unaffected exons are generated from exons not modulated following *ERG* knockdown (FDR > 0.5, maxPSI > 0.15 and minPSI < 0.85). (**C**) Numbers of significantly differentially spliced events identified after RBFOX2 knockdown in HeLa cells. ASEs: Alternatively spliced exons, RI: Retained intron, MXE: Mutually exclusive exons, A5SS: Alternative 5′ splice site, A3SS: Alternative 3′ splice site. (**D**) Overlap of significantly differentially spliced exons modulated upon ERG or RBFOX2 knockdown in HeLa cells. Expected overlap = 10. (**E**) Proportion of common target exons upon ERG and RBFOX2 inhibition shown in (D) (*n* = 132) categorized as ‘similar’ (blue sector) or ‘opposite’ (orange sector) depending on whether delta PSI values vary in respectively the same or opposite direction.

To investigate the mechanisms by which ERG regulates splicing, we performed motif enrichment analysis on our dataset of ERG-regulated ASEs in HeLa cells, using a compilation of 110 known RNA-binding protein (RBP) binding sites from the literature (41,42). Enrichment was scored relative to non-regulated exons. Motifs for RBPMS and for the RBFOX family stood out from this analysis as the first and second most significantly enriched motifs, respectively (Figure 3A). Interestingly, both RBPs are important splicing regulators for various developmental processes (43,44). We decided to focus our efforts on RBFOX factors, which have been extensively characterized as master splicing regulators. Enrichment of the RBFOX motif was the highest in proximal intronic sequences upstream of ERG-spliced-out ASEs, adding to the hypothesis that ERG controls alternative splicing through an RBFOX-dependent mechanism (Figure 3B).

To explore this possibility, we examined the effects of knocking-down *RBFOX2* on the mRNA splicing programs of HeLa cells. Among the RBFOX family, which include RBFOX1, RBFOX2 and the neuron-specific RBFOX3, RBFOX2 is the only member expressed in HeLa cells (Supplementary Figure S3A). Transfection with a *RBFOX2* siRNA resulted in efficient reduction of *RBFOX2* mRNA levels so as to reduce RBFOX2 protein levels to approximately 30% of normal, without inducing expression of *RBFOX1* or *RBFOX3* (Supplementary Figures S3B and S3C). In agreement with previous studies (45,46), knockdown of RBFOX2 mostly resulted in ASEs (55.5%, 399/719, Figure 3C). Out of 399 regulated ASEs, 233 were spliced-in and 166 were spliced-out in control cells as compared to RBFOX2-depleted cells (Supplementary Table S4). Comparison with our dataset of ERG-regulated ASEs revealed that a highly significant proportion (132/410; 32.2%, *P* < 10E–50) of ERG-regulated ASEs were also sensitive to RBFOX2 knockdown (Figure 3D). Strikingly, 96% of the ASEs regulated by both RBFOX2 and ERG were similarly regulated following knockdown of either of the two proteins (Figure 3E). Examples of independent or co-regulation by RBFOX2 and/or ERG were validated by RT-PCR analysis for a series of ASEs (Supplementary Figure S3D). Altogether, these data identify a large set of ASEs that are similarly regulated by both ERG and RBFOX2 in HeLa cells.

### ERG and FLI1 control a RBFOX2-dependent splicing program in HUVECs

To extend our findings, we repeated these analyses in human primary umbilical vein endothelial cells (HUVECs). HUVECs express both ERG and FLI1, which have been recognized as master regulators of endothelial gene expression programs (30). This cellular model thus offers the opportunity to compare ERG- and FLI1-mediated splicing effects in a biologically relevant context. We knocked down *ERG* and *FLI1* using specific siRNAs (Supplementary Figure S4A) and first analyzed gene expression changes. As excepted, knocking down ERG or FLI1 dramatically altered gene expression levels in HUVECs as we identified respectively 4212 (1607 up and 2605 down) and 3092 (1491 up and 1601 down) genes whose expression was significantly modified across three replicates (Supplementary Figure S4B, Supplementary Tables S5 and S6). As previously reported, ERG and FLI1 significantly co-regulated a large number of genes (Supplementary Figure S4C, D). Functional enrichment analysis revealed that ERG- and FLI1-regulated genes were mainly associated with GO terms related to cell cycle progression and chromosome segregation (Supplementary Figure S4E). All these results are fully consistent with previous reports and confirm the predominant roles of ERG and FLI1 in controlling the endothelial transcriptome (30).

**Figure 4.**
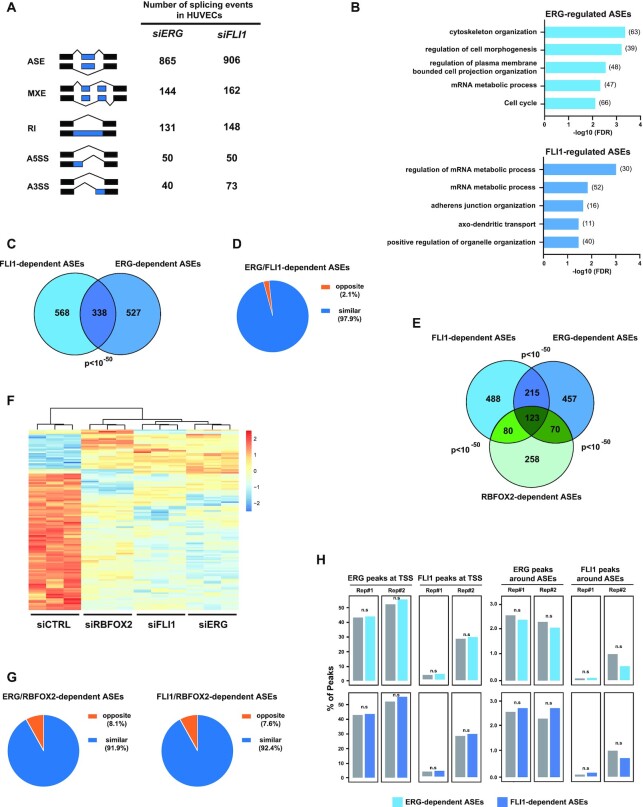
ERG and FLI1 shape the transcriptome at the level of gene expression and mRNA splicing in HUVECs. (**A**) Numbers of significantly differentially spliced events identified after *ERG* or FLI1 knockdown in HUVECs using rMATS with the following criteria: supported by at least 15 unique reads, |ΔPSI| > 10% and FDR < 0.05. ASE: Alternatively spliced exons, RI: Retained intron, MXE: Mutually exclusive exons, A5SS: Alternative 5′ splice site, A3SS: Alternative 3′ splice site. (**B**) Distribution of enriched GO biological process terms associated with differentially spliced genes (DSG, i.e. genes with at least one ERG-regulated ASE) following ERG (top) or FLI1 (bottom) knockdown in HUVECs. (**C**) Overlap of significantly differentially spliced exons modulated upon ERG or FLI1 knockdown in HUVECs. Expected overlap = 14. (**D**) Proportion of common target exons upon ERG or FLI1 inhibition shown in (C) (*n* = 338) categorized as ‘similar’ (blue sector) or ‘opposite’ (orange sector) depending on whether delta PSI values vary in respectively the same or opposite direction. (**E**) Overlap of significantly differentially spliced exons modulated upon ERG, FLI1 or RBFOX2 knockdown in HUVECs. Expected overlap: FLI1/ERG = 14, FLI1/RBFOX2 = 8, ERG/RBFOX2 = 8. (**F**) Heatmap of *Z*-scores of percent of spliced-in (PSI) values from common significantly differentially spliced exons between HUVEC cells transfected either with s*iERG*, *siFLI1* or *siRBFOX2*, relative to *siCTRL*. (**G**) Proportion of common target exons upon ERG and RBFOX2 or FLI1 and RBFOX2 inhibition in HUVECs shown in (**E**) (*n* = 193 and *n* = 203, respectively) categorized as ‘similar’ (blue sector) or ‘opposite’ (orange sector) depending on whether delta PSI values vary in respectively the same or opposite direction. (**H**) We compared genes with no ASEs (control group) and genes with ASEs (ERG-dependent in light blue and FLI1-dependent in dark blue). The barplots represent the proportion of genes that have a ERG or FLI1 ChIP peak on either TSS (left panel) or around exons (±250 bp, right panel). n.s: not significant, by chi-square test.

To confirm a role for ERG factors in alternative splicing regulation, we also profiled splicing changes in *ERG*- or *FLI1*-knocked down HUVEC cells. As observed in HeLa cells, the most frequent alternative splicing events observed in *siERG*-treated HUVEC cells were ASEs, with more than 865 ASEs being spliced-in (*n* = 667) or spliced-out (*n* = 198) in control versus ERG-depleted condition (Figure 4A, Supplementary Table S7). Similar observations were also made in *FLI1*-depleted HUVECs, where we detected 906 ASEs, 628 being more included and 278 more excluded in control cells (Figure 4A, Supplementary Table S8). GO analysis of DSGs revealed that ERG- and FLI1-dependent ASEs were found in genes associated with biological processes related to cell and plasma membrane morphogenesis, and adherens junction organization (Figure 4B). ERG and FLI1 co-regulated a significant number of ASEs (338 ASEs, Figure 4C). The effects of ERG or FLI1 on these common ASEs were almost systematically similar (i.e. they affected exon inclusion/exclusion in the same direction in 97.9% cases) (Figure 4D). Altogether, these observations confirm that ERG and FLI1 are key TFs shaping the endothelial transcriptome, but extend their functions beyond transcription, to alternative splicing regulation.

Our observations in HeLa cells suggested that ERG controls alternative splicing via a RBFOX2-related mechanism and independently of its DNA-binding activity. To extend these observations to HUVECs, we first compared RBFOX2-, ERG and FL1-dependent ASE changes. After knocking down RBFOX2 in HUVECs and profiling associated splicing changes, we identified 531 RBFOX2-dependent ASEs (Supplementary Table S9). Strikingly, 51,4% (273/531) of RBFOX2-dependent ASEs were also affected by depletion of *ERG* or *FLI1* (Figure 4E). Hierarchical clustering analysis (Figure 4F) and comparison of the direction of splicing regulation (i.e. spliced-in or spliced-out, Figure 4G) of the ASEs common to RBFOX2, ERG and FLI1 confirmed our observations in HeLa cells for ERG and RBFOX2, i.e. knocking down ERG or FLI1 recapitulated RBFOX2-depletion in most cases (91.9% and 92.4% for ERG and FLI1, respectively). These observations support a functional collaboration between ERG TFs and RBFOX2 in regulating pre-mRNA alternative splicing.

To gain further insights into the underlying mechanism, we tested whether the splicing function of ERG factors might be related to their transcriptional and DNA-binding ability. As observed in HeLa cells, we found no significant enrichment for ERG-regulated DEG among ERG-regulated DSG (Supplementary Figure S4F). In addition, analysis of publicly available ChIP-Seq datasets for ERG and FLI1 in HUVECs (30) revealed no significant enrichment of ERG or FLI1 peaks around the TSS of differentially spliced genes or 250 bp around ASEs (Figure 4H). We believe that these observations argue against a model where ERG or FLI1 predominantly affect pre-mRNA splicing via indirect mechanisms related to their DNA binding. They rather suggest that ERG factors control a RBFOX2-dependent splicing program, independently of their presence on DNA.

### ERG associates with RBFOX2 via its CTAD and is part of the RBFOX2-associated splicing complex LASR

**Figure 5.**
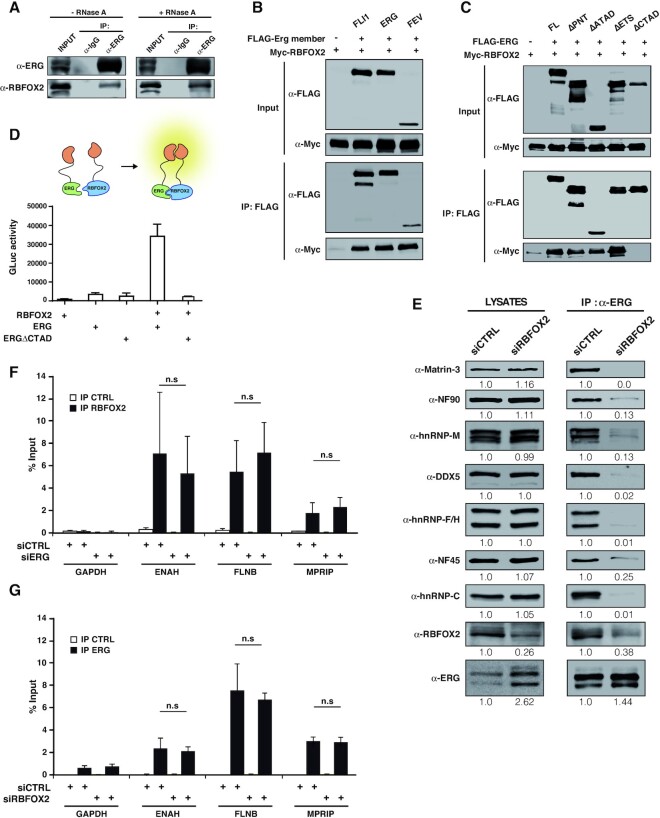
ERG physically associates with RBFOX2 through its C-terminal domain. (**A**) Western blot analysis of endogenous ERG immunoprecipitates from RNAse A-treated or untreated lysates from HeLa cells, using an RBFOX2 antibody. An anti-ERG antibody was used to control the immunoprecipitation efficiency. (**B**) Immunoprecipitation of FLAG-tagged ERG members (FLI1, ERG and FEV) and anti-Myc western blotting. Samples are lysates from HEK293 cells transfected with Myc-RBFOX2 alone or with either of the FLAG-tagged ERG members. (**C**) Immunoprecipitation of FLAG-tagged ERG deletion variants and anti-Myc and anti-FLAG western blotting. Samples are RNAse A-treated lysates from HEK293 cells transfected with Myc-RBFOX2 alone or with either of the FLAG-tagged ERG variants. (**D**) Protein interaction assay between RBFOX2 and ERG full-length or an ERG variant lacking the CTAD domain, using the *gaussia princeps* luciferase complementation method. Results are means ± s.e.m. from one representative experiment out of 3. (**E**) Immunoprecipitation of ERG followed by western blotting analysis using antibodies directed against the indicated components of the LASR complex. Samples are whole-lysates or anti-ERG immunoprecipitates from RNAse A-treated HeLa cell lysates transfected with control (*siCTRL*) or specific RBFOX2 siRNA (*siRBFOX2*). Quantification of the protein levels was performed by signal densitometry relatively to the *siCTRL* condition. (**F**) RNA-immunoprecipitations from HeLa cells transfected with control siRNA (*siCTRL*) or with siRNA against ERG (*siERG*) using control Ig (IP CTRL) or an antibody against RBFOX2 (IP RBFOX2). The presence of mRNA for GAPDH, ENAH, FLNB and MPRIP, all of which are subjected to ERG-dependent ASE was detected by RT-qPCR. Results are expressed as means±SD from five independent experiments, expressed relative to levels of the mRNAs in input. n.s.: not significant. (**G**) RNA-immunoprecipitations from HeLa cells transfected with control siRNA (*siCTRL*) or with siRNA against RBFOX2 (*siRBFOX2*) using control Ig (IP CTRL) or an antibody against ERG (IP ERG). The presence of mRNA for GAPDH, ENAH, FLNB and MPRIP was analyzed as described in (E).

Knocking-down *ERG* had no impact on RBFOX2 expression (Supplementary Figure S5A), ruling out the trivial explanation that ERG might indirectly participate in RBFOX2-dependent splicing regulation by controlling the expression level of RBFOX2. Next, we tested whether both proteins may be found in the same complex. In HeLa cells, we confirmed that endogenous RBFOX2 readily co-immunoprecipitates with endogenous ERG in a RNase-insensitive manner (Figure 5A). To extend these observations to other members of the families, we conducted co-immunoprecipitation experiments using exogenously expressed FLAG-tagged ERG family members and HA-tagged RBFOX1 or Myc-tagged RBFOX2 in HEK293 cells. Endogenously, these cells express no detectable levels of ERG family proteins and only very low levels of endogenous RBFOX2. Anti-FLAG immunoprecipitation followed by western blot analysis revealed that HA-RBFOX1 (Supplementary Figure S5B) and Myc-RBFOX2 (Figure 5B) co-immunoprecipitate with ERG, FLI1 and FEV FLAG-tagged constructs. Using FLAG-tagged ERG variants lacking individual domains to identify the RBFOX2-interacting region of ERG, we found that only the ERG variant lacking the CTAD region, shown above to be important for the splicing activity of ERG in the *SMN2* reporter assay, had lost the ability to associate with RBFOX2 (Figures 2H and 5C). Protein interaction assays based on complementation of the *Gaussia* luciferase (gPCA) (47) confirmed that the CTAD is required for association with RBFOX2 and demonstrated that both proteins are in close proximity *in vivo* (Figure 5D). These data demonstrate that ERG family proteins associate with RBFOX2 through their conserved CTAD domain (Supplementary Figure S2C).

Recently, the splicing activity of RBFOX proteins was shown to rely on their association with a large complex of 8 RBPs (Matrin-3, NF90, hnRNP M, DDX5, hnRNP F/H, NF45 and hnRNP C) called LASR (Large Assembly of Splicing Regulators) (37). Interestingly, five components of LASR (DDX5, hnRNP M, hnRNP F/H, hnRNP C) are found among the 26 splicing-related ERG partners in protein-protein interaction databases (Supplementary Table S3). In agreement with this, we found that endogenous ERG co-immunoprecipitates with all LASR components (Figure 5E). Importantly, the co-immunoprecipitation of LASR components with ERG was dramatically reduced in RBFOX2-depleted cells, albeit to different extents, suggesting that RBFOX2 is essential to bridge ERG and LASR.

We then looked at ERG and RBFOX2 association with three pre-mRNAs (*ENAH*, *FLNB* and *MPRIP*) that were similarly regulated by both factors and have an RBFOX consensus motif in their vicinity. Using RNA-immunoprecipitation experiments, we confirmed that RBFOX2 specifically associates with these pre-mRNAs, but not with the non-regulated GAPDH pre-mRNA (Figure 5F). RBFOX2 association with the three target pre-mRNAs was independent of the presence of ERG. Interestingly, ERG was also associated with these three target pre-mRNAs, but not with the GAPDH pre-mRNA (Figure 5G). Because our RNA-immunoprecipitation experiments were done on formaldehyde-crosslinked cells, these data demonstrate that ERG is in a complex with its target pre-mRNAs, either due to direct RNA binding or through an intermediate protein. The association of ERG with its target pre-mRNAs was not affected by RBFOX2 depletion (Figure 5G), suggesting that it is not dependent on RBFOX2.

Altogether, our data suggest a model, where the similar effects of ERG and RBFOX2 on the inclusion of specific exons in pre-mRNAs rely on (i) the association of both factors with their common target pre-mRNAs, independently of each other; and (ii) their association, with RBFOX2 recruiting ERG to functional RBFOX2/LASR complexes.

### EWS-FLI1 and RBFOX2 are part of the same complex and converge on a splicing program in Ewing sarcoma cells

**Figure 6.**
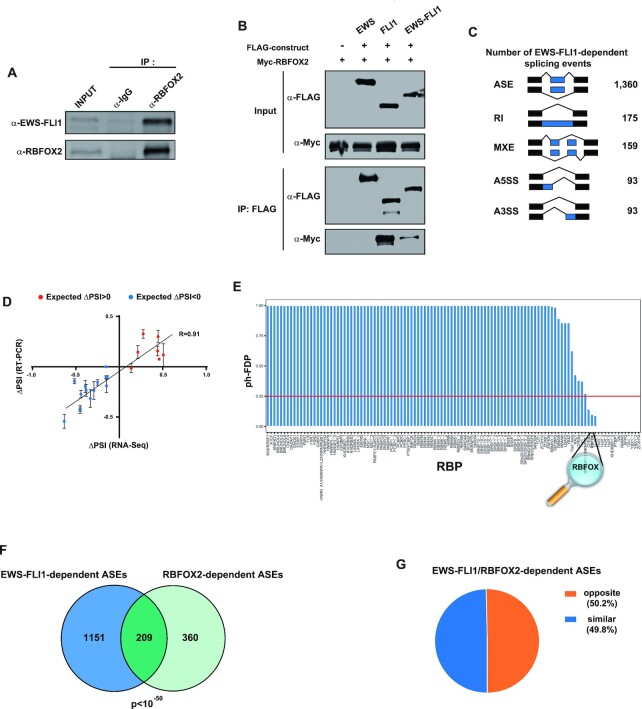
EWS-FLI1 fusion protein associates with RBFOX2 and controls a common splicing program. (**A**) Immunoprecipitation of endogenous RBFOX2 followed by anti-EWS-FLI1 and anti-RBFOX2 western blotting. Samples are control (IgG) and anti-RBFOX2 immunoprecipitates from A673/TR/shEF cells. An anti-RBFOX2 antibody was used to control the immunoprecipitation efficiency. (**B**) Immunoprecipitation of FLAG-tagged EWS, FLI1 or EWS-FLI1 and anti-Flag and anti-Myc western blotting. Samples are RNAse A-treated lysates from HEK293 cells transfected with Myc-RBFOX2 together with the FLAG empty vector or with FLAG-tagged EWS, FLI1 or EWS-FLI1. (**C**) Numbers of significantly differentially spliced events identified by rMATS after EWS-FLI1 inhibition in A673/TR/shEF cells. ASE: Alternatively spliced exons, RI: Retained intron, MXE: Mutually exclusive exons, A5SS: Alternative 5′ splice site, A3SS: Alternative 3′ splice site. (**D**) Correlation of ΔPSI values analyzed by RT-PCR or RNA-seq from a subset of exons showing differential splicing between A673/TR/shEF cells at day0 and day7 of doxycycline treatment. (**E**) Post-hoc false discovery proportion (ph-FDP) values of motif enrichment analysis performed on EWS-FLI1-regulated spliced-in exons in A673/TR/shEF cells. Red line represents the upper-bound of the significant RBPs at 25% FDP. (**F**) Overlap between EWS-FLI1- and RBFOX2-dependent exons in A673/TR/shEF cells. Expected overlap = 22. (**G**) Proportion of common target exons upon EWS-FLI1 and RBFOX2 inhibition shown in (F) (*n* = 209) categorized as ‘similar’ (blue sector) or ‘opposite’ (orange sector) depending on whether delta PSI values vary in respectively the same or opposite direction.

While EWS-FLI1 is known to affect a large-scale splicing program in Ewing sarcoma cells (11), its function in splicing has been assumed to rely on its EWS moiety (10). Interestingly, our results suggest that the FLI1-derived (C-terminal) moiety might contribute to EWS-FLI1 splicing function. To test this hypothesis, we first evaluated whether EWS-FLI1 associates with RBFOX2 in Ewing sarcoma cells. Co-immunoprecipitation experiments in A673/TR/shEF (20), a well-characterized Ewing sarcoma cell line, revealed the presence of EWS-FLI1 in endogenous RBFOX2 complexes (Figure 6A). This association was resistant to RNase A treatment and mediated by the FLI1 moiety, as it was observed for both FLI1 and EWS-FLI1, but not for EWS (Figure 6B). The demonstration of an association between EWS-FLI1 and RBFOX2 prompted us to further investigate the potential role of RBFOX2 in EWS-FLI1-mediated splicing regulation.

First, we identified the set of alternative splicing events regulated by EWS-FLI1 using the A673/TR/shEF cell line that harbors a doxycycline-inducible shRNA targeting the *EWS-FLI1* fusion transcript (20). RNA-seq was performed after seven days of doxycycline (DOX) treatment (d7), which leads to a robust depletion of EWS-FLI1 (Supplementary Figure S6A). Alternative splicing analysis revealed that knockdown of EWS-FLI1 mostly resulted in ASEs (72.3%, 1360/1880, Figure 6C and Supplementary Table S10). Among these ASEs, we validated a set of 20 events by RT-PCR (Figure 6D). We also performed RNAseq experiments 10 days (d17) and 15 days (d22) after washing out DOX from the media to re-allow EWS-FLI1 expression (Supplementary Figure S6B). We observed that most ASEs were reverted to their basal levels 10 days later (d17), when EWS-FLI1 expression was fully restored (Supplementary Figure S6C). This experiment shows that detected ASEs are fully reversible and strongly correlate with EWS-FLI1 expression level.

Among EWS-FLI1-dependent ASEs, we then looked for potential RBP motifs upstream or downstream of regulated exons. As previously observed for ERG-dependent exons in HeLa cells, we found a significant enrichment of the RBFOX-binding motif around EWS-FLI1-regulated exons (Figure 6E). To further support a functional link between EWS-FLI1 and RBFOX2, we analyzed splicing changes following RBFOX2 depletion in A673/TR/shEF Ewing sarcoma cells (Supplementary Figure S6D). Knockdown of *RBFOX2* was associated with 768 splicing events. As observed in HeLa and HUVEC cells, ASEs accounted for the majority of splicing events regulated in Ewing sarcoma cells following *RBFOX2* knockdown (74,1%, 569/768) (Supplementary Figure S6E, Supplementary Table S11). Consistent with the enrichment of the RBFOX-binding motif around EWS-FLI1-regulated exons, comparison of EWS-FLI1- and RBFOX2-regulated ASEs revealed a highly significant subset of common targets, as EWS-FLI1 regulated more than one third (36.7% 209/569) of RBFOX2-dependent ASEs (Figure 6F). However, in striking contrast with what we observed for wild-type FLI1 (and ERG), depletion of EWS-FLI1 or RBFOX2 did not systematically have similar effects on common ASEs. Instead, knocking down EWS-FLI1 had an opposite effect to that of knocking down RBFOX2 in 50.2%, (105/209) of cases (Figure 6G). Altogether, these data indicate that EWS-FLI1 and RBFOX2 interact with each other, and regulate a common set of ASEs in Ewing sarcoma cells, either in a similar or opposite manner.

### *ADD3* splicing induced by EWS-FLI1 is a phenotypic-driver in Ewing sarcoma

**Figure 7.**
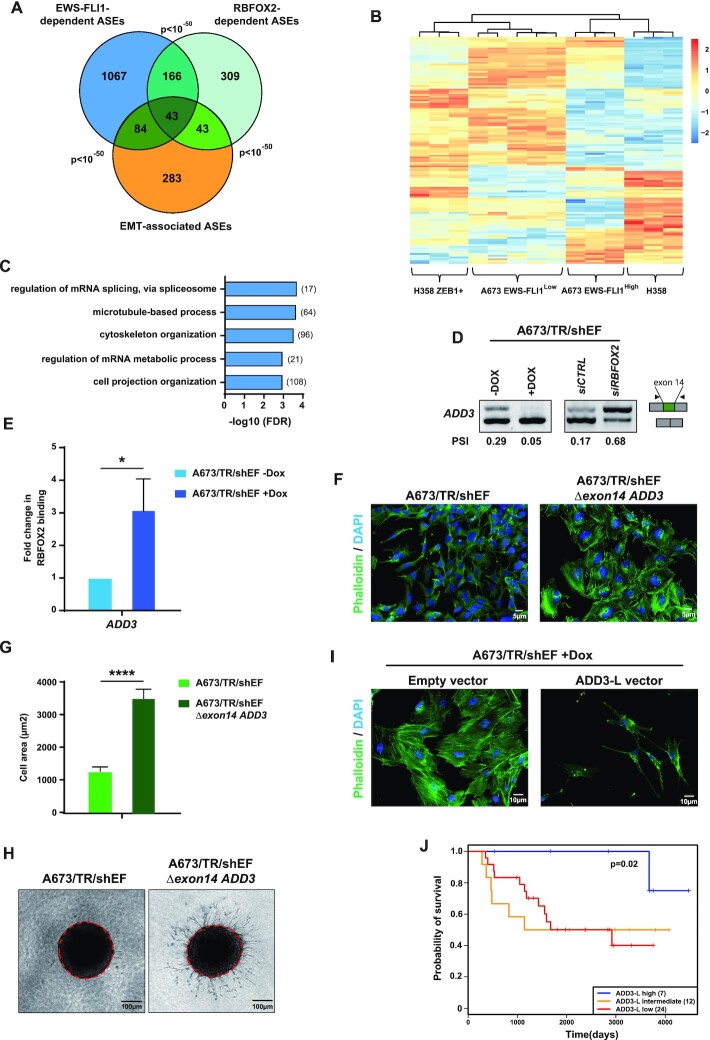
ADD3 pre-mRNA splicing induced by EWS-FLI1 participates in Ewing sarcoma phenotype. (**A**) Overlap between EWS-FLI1- or RBFOX2-dependent exons in A673/TR/shEF cells and EMT-associated splicing events in H358 human epithelial cells. All pair-wise comparisons are highly significant (*P*-value < 10^–50^). (**B**) Heatmap of *Z*-scores of percent of spliced-in (PSI) values from significantly differentially spliced exons in EWS-FLI1^high^ (A673/TR/shEF cells without Dox treatment) versus EWS-FLI1^low^ (A673/TR/shEF cells at day 7 of Dox treatment) and in epithelial versus mesenchymal-like ZEB1-expressing H358 cells. (**C**) Distribution of top significant GO biological process terms from genes containing EWS-FLI1 and RBFOX2-regulated exons. Number of spliced genes regulated by EWS-FLI1 and RBFOX2 in each GO are indicated in brackets. (**D**) RT- PCR analysis of *ADD3* exon 14 splicing. Samples are RNA from A673/TR/shEF cells expressing (-DOX) or not (+DOX) EWS-FLI1 (left) and RNA from A673/TR/shEF cells expressing EWS-FLI1 and transfected with control siRNA (*siCTRL*) or a specific RBFOX2 siRNA (*siRBFOX2*). (**E**) RNA-immunoprecipitation experiments using anti-RBFOX2 antibodies followed by RT-qPCR to detect *ADD3* transcripts. Samples are RNA from A673/TR/shEF cells expressing (–DOX) or not (+DOX) EWS-FLI1. Results shown are means ± s.e.m. (*n* = 3 independent experiments) relative to the –DOX condition. *P < 0.05 by two-tailed unpaired Student's *t*-test. (**F**) Immunofluorescence of actin filaments stained with phalloidin (green channel) and DAPI (blue channel) of A673/TR/shEF cells and cells deleted for *ADD3* exon 14 genomic region (A673/TR/shEF *Δexon14 ADD3*). (**G**) Measurement of cell area of A673/TR/shEF cells and cells deleted for *ADD3* exon 14 genomic region (A673/TR/shEF *Δexon14 ADD3*). Data are represented as mean ± s.e.m. (*n* > 100 cells). ****P < 0.0001. (**H**) Three-dimensional type-I collagen multicellular spheroid invasion assay of A673/TR/shEF cells and cells deleted for *ADD3* exon 14 genomic region (A673/TR/shEF *Δexon14 ADD3*). Red dotted lines represent the initial spheroid area. (**I**) Immunofluorescence of actin filaments stained with phalloidin (green channel) and DAPI (blue channel) of A673/TR/shEF cells treated with DOX for 7 days (EWS-FLI1^low^ expressing cells) and transfected with either empty vector or vector expressing the ADD3-L isoform. (**J**) Kaplan-Meier curve of *ADD3* exon 14 PSI values in 43 Ewing tumors showing significant differences between tumors expressing high and low levels of ADD3-L isoform. Tumors were separated by k-means clustering. Number of tumors in each subgroup is indicated in brackets.

RBFOX2 is a key regulator of mesenchymal-specific splicing programs that promote the mesenchymal phenotype (48,49). In contrast, EWS-FLI1 is considered as a repressor of mesenchymal features in Ewing sarcoma cells (50–52). Based on our observation that EWS-FLI1 represses a large subset of RBFOX2-regulated ASEs, we thus hypothesized that EWS-FLI1 might inhibit specific RBFOX2-dependent ASEs to antagonize mesenchymal features in Ewing sarcoma cells. Alternative splicing events associated with EMT have been recently explored by induction of the EMT promoting factor ZEB1 in lung carcinoma H358 cells (53). Despite the different cellular backgrounds, we found a highly significant overlap between EWS-FLI1-regulated ASEs, RBFOX2-regulated ASEs, and ASEs associated with EMT (Figure 7A). Hierarchical clustering illustrated similarities between the splicing landscapes of *EWS-FLI1*-knocked-down A673/TR/shEF cells and of ZEB1-expressing H358 cells, while normal H358 clustered with control A673/TR/shEF cells (Figure 7B), consistent with the idea that EWS-FLI1 is a repressor of mesenchymal features. This indicates that decreasing expression of EWS-FLI1 in Ewing sarcoma cells induces a mesenchymal splicing program, in addition to a mesenchymal gene expression program as previously reported (50–52). Accordingly, functional analysis revealed that common ASEs of EWS-FLI1 and RBFOX2 (*n* = 209, Supplementary Table S12) are enriched in genes linked to cytoskeleton and membrane organization (Figure 7C).

We were particularly interested in genes containing ASEs that were antagonistically regulated by EWS-FLI1 and RBFOX2, and turned our attention towards the *ADD3* gene, which encodes an EMT-associated protein playing a role in actin cytoskeleton remodeling (18). By RT-PCR, we verified that EWS-FLI1 promotes the *ADD3-L* isoform, which contains exon 14, whereas RBFOX2 reduces the inclusion of exon 14 of *ADD3* in Ewing sarcoma cells (Figure 7D). We further validated the regulation of *ADD3* exon 14 splicing by EWS-FLI1 depletion using siRNA transfection in a second Ewing sarcoma cell line (MHH-ES1, Supplementary Figure S7A). RNA-seq analysis of a collection of 43 Ewing tumors showed that the relative inclusion levels of *ADD3* exon 14 were positively correlated with *EWS-FLI1* expression levels (*P* = 0.017; *R* = 0.39) (Supplementary Figure S7B).

To further understand how EWS-FLI1 might antagonize RBFOX2-dependent splicing of *ADD3*, we first examined RBFOX2 expression in *EWS-FLI1*-expressing *versus* -knocked down A673/TR/shEF cells. We found that *RBFOX2* mRNA expression levels did not change following DOX-mediated downregulation of *EWS-FLI1* (Supplementary Figure S7C), thus excluding the trivial explanation that EWS-FLI1 might repress *RBFOX2* expression. Next, because a potential RBFOX2-binding motif is found near *ADD3* exon 14, we performed RNA-immunoprecipitation experiments of endogenous RBFOX2 in the A673/TR/shEF cell line upon DOX treatment. Quantitative RT-PCR analysis revealed that the association of RBFOX2 with *ADD3* pre-mRNA near exon 14 was strongly decreased in the presence of EWS-FLI1 (Figure 7E). These data suggest that EWS-FLI1 promotes *ADD3* exon 14 inclusion and ADD3-L isoform production by preventing RBFOX2 binding to *ADD3* pre-mRNA.

Depletion of EWS-FLI1 induces a switch towards a mesenchymal phenotype, with cells displaying increased actin stress fibers, cell size, and invasion capacity (50). Interestingly, preventing the expression of the ADD3-L isoform in EWS-FLI1 expressing cells, either by using a specific siRNA targeting exon 14 in A673/TR/shEF and MHH-ES1 cell lines (Supplementary Figure S7D) or by removing this exon using a CRISPR/Cas9 approach in A673/TR/shEF cells (Supplementary Figure S7E–G), led to increased actin stress fibers (Figure 7F and Supplementary Figure S7H). Further analysis of cells with CRISPR deletion of *ADD3* exon 14 showed an increase in cell size and invasion capacity (Figure 7G and H). Conversely, in DOX-treated A673/TR/shEF cells, which express low levels of EWS-FLI1 and therefore of *ADD3-L* (See Figure 7D), ectopic expression of the ADD3-L isoform reduced stress fibers formation (Figure 7I and Supplementary Figure S7I). These observations strongly suggest that the ADD3-L isoform contributes to the repression of mesenchymal features by EWS-FLI1 in Ewing sarcoma cells.

Finally, we investigated the prognostic significance of *ADD3-L* mRNA expression in patients with Ewing sarcoma. We generated Kaplan-Meier curves by stratifying patients into 3 groups using k-means clustering: tumors with high, intermediate and low inclusion of *ADD3* exon 14. Patients with high inclusion levels of *ADD3* exon 14 showed a significantly better survival than patients with low inclusion levels (Figure 7J). This observation supports the hypothesis that high levels of the ADD3-L isoform in tumors repress the mesenchymal phenotype of Ewing sarcoma cells hence decreasing their metastatic potential. Altogether, these data strongly suggest that the regulation of *ADD3* splicing by EWS-FLI1 is important for Ewing sarcoma biology.

## DISCUSSION

TFs are traditionally defined as sequence-specific DNA-binding proteins controlling transcription initiation. Although mounting evidence indicates that they can also participate in downstream mRNA processing events, in particular splicing, the prevailing model suggests that they do so via indirect mechanisms (15). These include (i) modification of RNA polymerase II elongation rate, which modulates splicing by altering the kinetics of exposure of splice sites; (ii) recruitment of transcriptional coactivators that affect splicing and (iii) modulation of the expression of direct splicing regulators (16). More recently, it was shown that some TFs can bind directly to pre-mRNA and control alternative splicing via unknown but direct mechanisms (17). Here, we report that the transcription factor ERG controls hundreds of alternative splicing events. Although some of these events might be indirectly regulated by ERG (*i.e*. if ERG controls the expression levels of splicing regulators), our data strongly argue in favor of a direct role for ERG in pre-mRNA splicing. First, ERG TFs are found in association with nascent pre-mRNA. Second, a splicing minigene assay shows that ERG TFs specifically affect inclusion rates of the cassette exon only when tethered to the reporter transcript. Third, ERG co-immunoprecipitates with the splicing regulator RBFOX2 and its key functional partners within the LASR splicing complex, which is required for the splicing activity of RBFOX2 (37). Fourth, knockdown experiments demonstrate that ERG TFs and RBFOX2 control a common set of ASEs with an enrichment in RBFOX2 binding motifs in the preceding intron; it is thus likely that ERG-dependent splicing relies on the recognition of these motifs by RBFOX2. Fifth, in addition to LASR, ERG engages in a variety of interactions with core spliceosome constituents, such as SF3A1, U1C, U1–70K and U2AF65 (see Figure 2D). Strikingly, RBFOX1/2 have also been shown to either interact or interfere with these proteins (54,55). Sixth, our findings suggest that the splicing function of ERG is independent of its DNA-binding and transcriptional activity. Indeed, there was no significant overlap between differentially expressed and differentially spliced genes in ERG knockdown cells; there was no enrichment of ERG binding sites in DNA regions around ERG regulated exons and the TSS of corresponding genes; and a transcriptionally inactive ERG variant lacking the DNA-binding domain (40) exhibited full splicing activity in our minigene assay. Finally, further analyses on exons co-regulated by both ERG and RBFOX2 indicate that both factors associate (directly or indirectly) with pre-mRNAs around these exons, independently of each other.

While the enrichment of RBFOX2-binding sites around ERG-regulated exons raised the possibility that RBFOX2 might recruit ERG onto pre-mRNA, this model is ruled out by the fact that ERG association with its target pre-mRNAs is independent of RBFOX2. Conversely, a model where DNA-bound ERG might recruit RBFOX2 to ERG target genes and promote its binding to its RNA recognition motifs around alternative exons, is ruled out by the lack of enrichment of ERG binding sites in DNA regions around ERG regulated exons and the TSS of corresponding genes, and by the fact that ERG depletion does not affect RBFOX2 association with target pre-mRNAs. Importantly, ERG incorporation into the RBFOX2/LASR complex is largely dependent on RBFOX2. Altogether, our data suggest a model, where pre-mRNA-bound RBFOX2 is not responsible for bridging ERG onto its target pre-mRNAs, but to the LASR complex. Because we found that ERG also associates with several components of the spliceosome, its presence within LASR might affect the activity and/or the composition of the splicing complex around regulated exons or alter the interaction of the RBFOX2/LASR complex with the splicing machinery. The network of interactions between ERG, RBFOX2/LASR, spliceosome core components and potential additional RBPs, and the dynamic regulation of these interactions are very exciting issues that deserve to be further investigated. Beyond ERG, our observations provide support to the idea that alternative splicing regulation is a complex process governed by a dynamic protein network including spliceosomal proteins and RBPs but also TFs (17,56). Our work also adds to a growing body of evidence showing that TFs are multitasking proteins, orchestrating multiple aspects of mRNA biogenesis.

The overlap between ERG- and RBFOX2-regulated ASEs is not total, suggesting that both proteins can also regulate alternative splicing independently of each other. Along these lines, we found that ERG members, through their CTAD, co-immunoprecipitate with RBPMS (RNA-binding protein with multiple splicing) and QKI (Quaking Homolog, KH Domain RNA Binding) ((3) and unpublished observations), two splicing regulators whose binding motifs were significantly enriched around ERG- or EWS-FLI1-dependent exons, respectively. Like RBFOX2, QKI and RBPMS regulate alternative splicing during cell differentiation (58–60), and QKI promotes mesenchymal splicing patterns (53). On these grounds, it would be interesting to test the possibility of a functional cooperation between ERG TFs and other RBPs, including RBPMS and QKI.

Perturbation of alternative splicing programs is a feature of Ewing sarcoma, and has been attributed to the presence of oncogenic fusion proteins (e.g. EWS-FLI1) (10–12) However, while EWS-FLI1 function in splicing was thought to be due to its EWS moiety, our findings demonstrate that the FLI1-derived moiety is also a major contributor. In sharp contrast to the convergent effects shared by RBFOX2 and wild-type ERG family proteins on their common set of ASEs, the EWS-FLI1 fusion antagonizes a large proportion of RBFOX2-dependent ASEs. The observation that RBFOX2 binding to its target pre-mRNA, *ADD3* is increased after depletion of EWS-FLI1 in Ewing sarcoma cells, suggests that EWS-FLI1 expression may interfere with RNA binding by RBFOX2. Splicing regulation often requires condensation of cooperative splicing factors into phase-separated complexes via aggregation of low-complexity (LC) or intrinsically discorded (ID) domains commonly found in RBPs (61). The C-terminal domain of RBFOX2, contains a LC/ID region that mediates higher-order assembly of RBFOX/LASR complexes and is required for its splicing activity (37,62). Interestingly, EWS-FLI1 also contains a LC region derived from EWS and can participate in phase transition processes (7,63). Thus, EWS-FLI1 might divert RBFOX2 away from the LASR assemblies through competing phase transition processes. Alternatively, interaction with EWS-FLI1 may modify the affinity of RBFOX2 for its binding RNA motif. However, on a large set of ASEs, EWS-FLI1 and RBFOX2 regulate alternative splicing in the same direction. This indicates that EWS-FLI1 has ambivalent functions on RBFOX2-regulated ASEs, which may depend on context, such as nearby binding of additional RBPs (e.g. QKI or RBPMS) or the exact composition of the LASR complex. In this regard, it is interesting to mention that EWS-ETS fusion proteins have been shown to have gain-of-function activities on some DNA target sites such as microsatellites but also to demonstrate dominant negative action toward wild-type ETS protein on other DNA binding sites (64,65). Even though expression levels of wild-type ERG members are not detected in Ewing sarcoma cells, we cannot exclude that EWS-FLI1 may interfere with the potential splicing activity of other members of the ETS family that may be involved in splicing as well, beyond the ERG subfamily proteins. In this respect, it is interesting to mention that PU1/Spi1 has been shown to interact directly with RNA and to modulate splicing (66).

While the idea of EWS-FLI1 influencing alternative splicing was expressed almost two decades ago (12), the only functional relevance of such a function for the Ewing sarcoma oncogenic process was a splicing regulation of the *ARID1A* gene (67). Here, we show that EWS-FLI1 interferes with the function of RBFOX2, a factor that promotes EMT-specific (mesenchymal) splicing programs (68). A number of publications support a mesenchymal-stem-cell origin for Ewing cells (52,69). Our study indicates that the EWS-FLI1-induced reprogramming of these cells may not only rely on transcriptional effects, in particular via genome-wide activation of GGAA microsatellites (7,8,70) but also on post-transcriptional effects, such as modulation of the pro-mesenchymal splicing program driven by RBFOX2. Cell-to-cell variation of the expression level of EWS-FLI1 has been recently involved in the plasticity of Ewing sarcoma cells (51). The hypothesis of the alteration of splicing programs by EWS-FLI1 participating in this plasticity process can now be thoroughly tested. Regarding this hypothesis, we show that the EWS-FLI1-induced exon 14-containing isoform of ADD3, ADD3-L contributes to the repression of the mesenchymal phenotype in Ewing sarcoma cells. Specific targeting of the ADD3-L isoform leads to an increased ability of Ewing sarcoma cells to migrate. Further experiments are needed to decode the precise function of the ADD3 domain encoded by exon 14, especially in the assembly of the spectrin-actin network. The observation, which needs to be confirmed on an independent series, that high levels of the ADD3-L isoform in tumors are associated with increased survival rates of patients, suggests that it may constitute an interesting prognosis biomarker.

The widespread effects of wild-type ERG family proteins on alternative splicing, that we identified in this study in HeLa and HUVEC cells, are enriched in gene functions that are relevant to the phenotypic effects of these proteins (e.g. mitosis in HeLa cells, cell morphology and adherens junctions in endothelial cells). In the future, the role of ERG-mediated splicing regulation deserves to be investigated, in these cells as well as in additional context where these proteins are involved (e.g. prostate cancer cells). Finally, our work suggests that alternative splicing regulation by transcription factors may be an underappreciated phenomenon.

## DATA AVAILABILITY

The datasets generated in this study have been deposited to the NCBI repository (PRJNA521683).

## Supplementary Material

gkab305_Supplemental_Files

## Notes

Present address: Olivier Saulnier, The Arthur and Sonia Labatt Brain Tumor Research Center, The Hospital for Sick Children, Toronto, ON M5G 0A4, Canada.

Present address: Olivier Saulnier, Developmental & Stem Cell Biology Program, The Hospital for Sick Children, Toronto, ON M5G 0A4, Canada.

Present address: Marie-Ming Aynaud, Lunenfeld-Tanenbaum Research Institute, Mount Sinai, M5G 1X5, Toronto, ON, Canada.

Present address: Xavier Rambout, Department of Biochemistry and Biophysics, School of Medicine and Dentistry, Center for RNA Biology, University of Rochester, Rochester, NY 14642, USA.
