# Supplementary material for: ERG transcription factors have a splicing regulatory function involving RBFOX2 that is altered in the EWS-FLI1 oncogenic fusion
Source: Nucleic Acids Res. 2021 May 1;49(9):5038–56. doi: 10.1093/nar/gkab305 (PMC8136815; doi:10.1093/nar/gkab305)
Supplement: gkab305_Supplemental_Files [file gkab305_supplemental_files.zip › Supplementary_Figures_and_Legends.pdf]

Supplementary Figures Legends

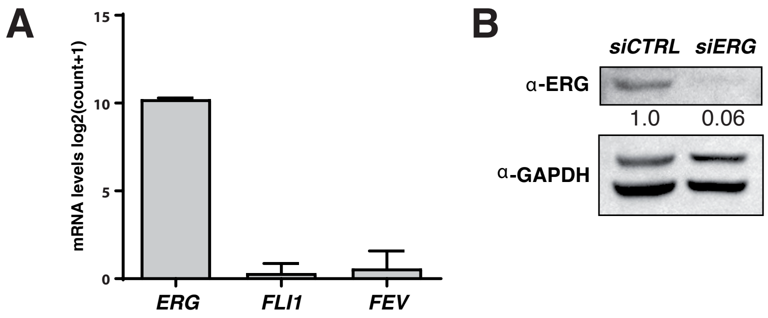

Supplementary Figure S1

Supplementary Figure S1: (A) mRNA expression levels of *ERG*, *FLI1* and *FEV* in HeLa cells from RNA-seq analysis. (B) Western blotting of ERG in HeLa cells following transfection with control (*siCTRL*) or *ERG*-specific siRNA (*siERG*). GAPDH was used as loading control. Quantification of the ERG/GAPDH ratio was performed by signal densitometry relatively to the *siCTRL* condition.

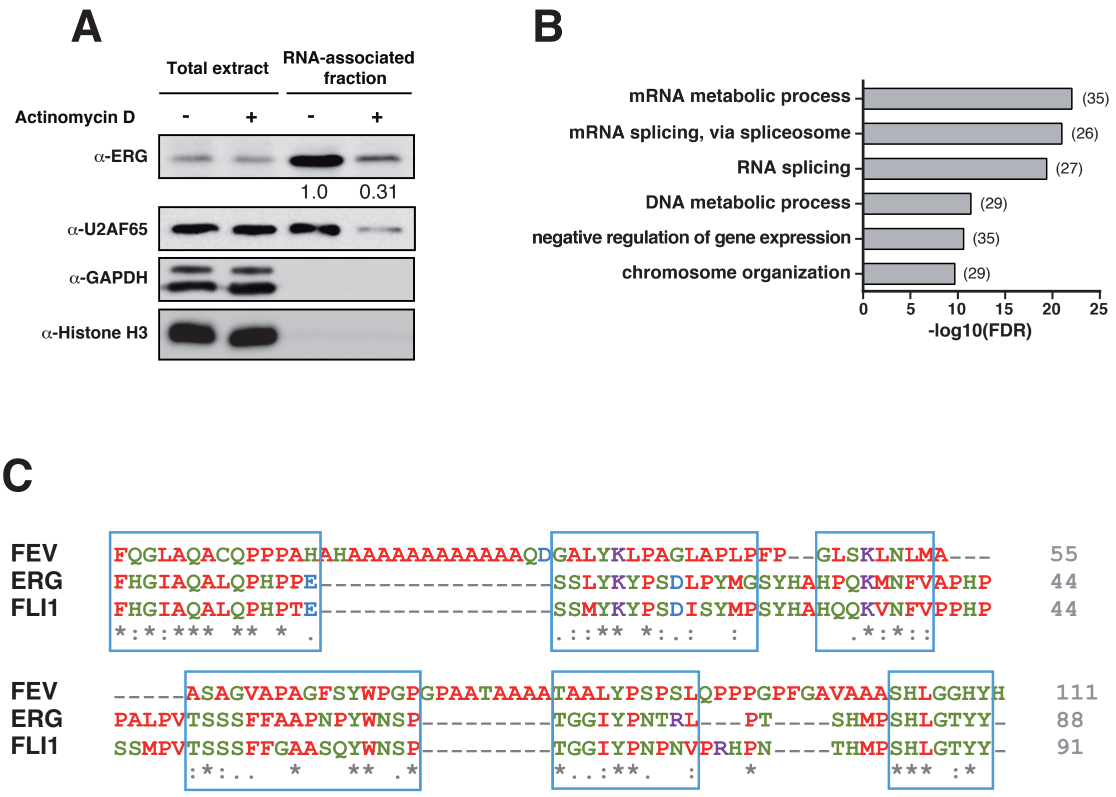

Supplementary Figure S2

Supplementary Figure S2: (A) Immunoblot analysis of ERG in total and RNA-associated HMW fraction from HeLa cells. U2AF65, GAPDH and Histone H3 specific antibodies were used as control for fraction purity. Samples are lysates from HeLa cells treated (+) or not (-) with 25 µM Actinomycin D for 2h. (B) Distribution of the most significantly enriched GO molecular function terms from a curated list of 97 proteins known as ERG-interactors (from the BioGRID and STRING databases). Number of ERG-interactors in each GO are indicated in brackets. (C) Alignment of the CTAD regions of FEV, ERG and FLI-1. Regions of high homology are boxed in blue.

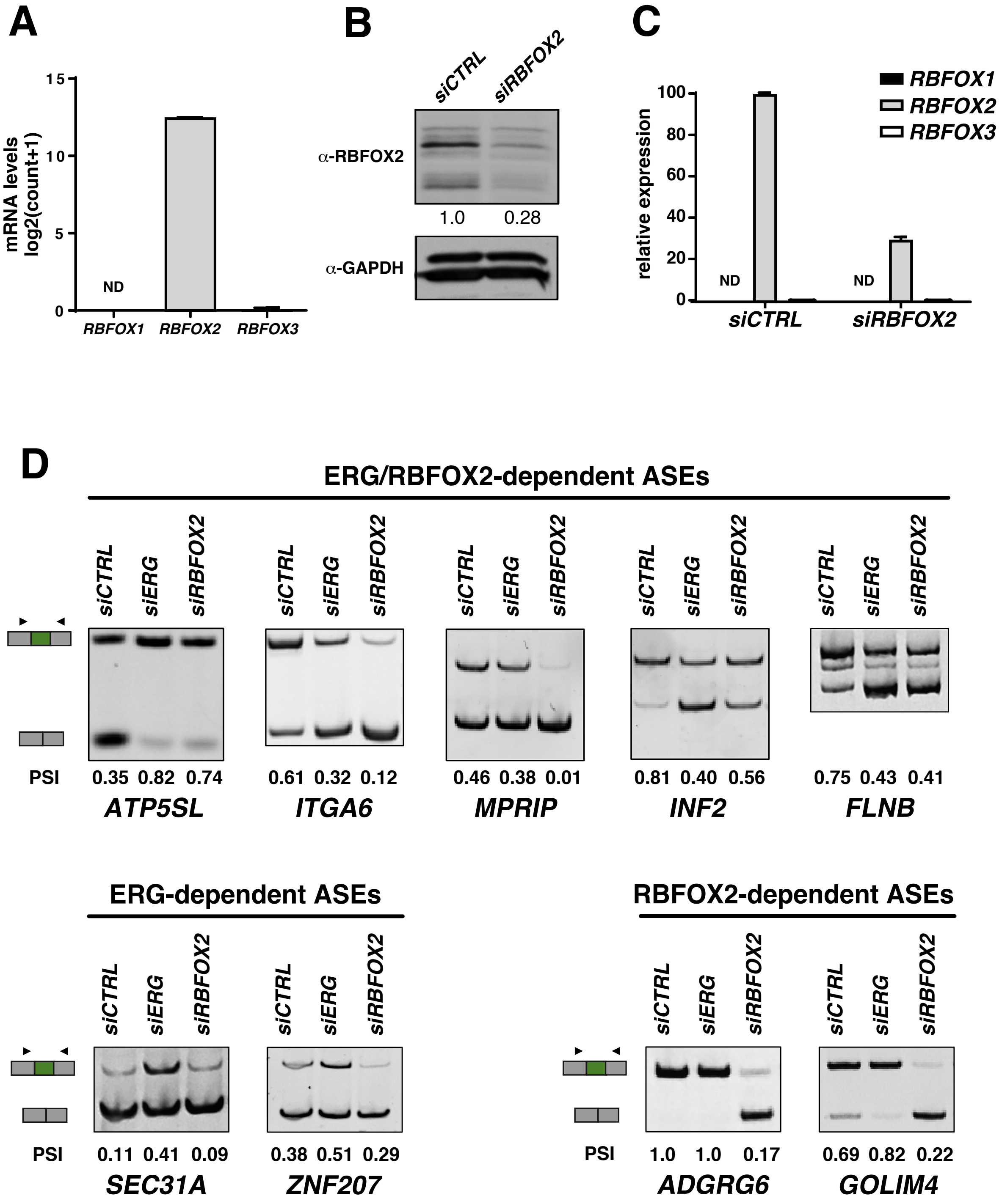

Supplementary Figure S3

Supplementary Figure S3: (A) mRNA expression levels of *RBFOX1*, *RBFOX2*, *RBFOX3* in HeLa cells from RNA-seq analysis. ND: Non-Detected. (B) Western blotting of RBFOX2 and GAPDH, as control. Samples are total lysates from HeLa cells transfected with control (*siCTRL*) or specific *RBFOX2* siRNA (*siRBFOX2*). Quantification of the RBFOX2/GAPDH ratio was performed by signal densitometry relatively to the *siCTL* condition and is indicated. (C) mRNA expression levels of *RBFOX1*, *RBFOX2*, *RBFOX3* in HeLa cells transfected either with control (siCTRL) or specific *RBFOX2* siRNA (*siRBFOX2*) from RNA-seq analysis. ND: Non-Detected. (D) RT-PCR analysis of representative ASEs co- or independently regulated by ERG and RBFOX2. Samples are RNA from HeLa cells transfected by control *siRNA* or *siRNA* against ERG or RBFOX2.

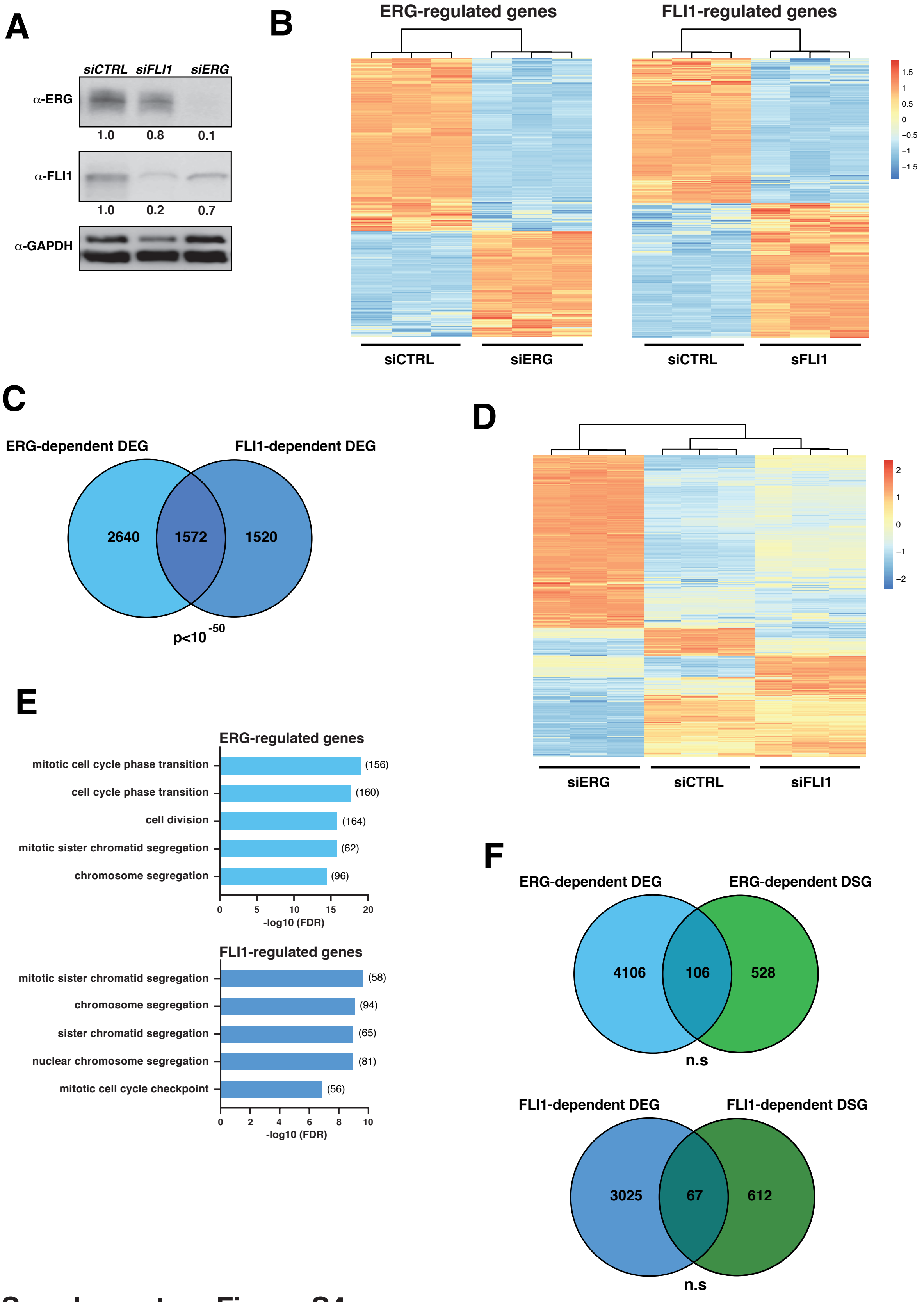

Supplementary Figure S4

Supplementary Figure S4: (A) Western blotting of ERG, FLI1 and GAPDH, as control. Samples are total lysates from HUVECs transfected with control (*siCTRL*) or specific siRNA against ERG (*siERG*) or FLI1 (*siFLI1*). (B) Heatmap of Z-scores of genes that are differentially expressed in HUVECs after transfection with s*iERG* (left map) or *siFLI1* (right map) relative to *siCTL*. Differentially expressed genes were identified using htseq-count (FDR<0.05 and FC>2). (C) Overlap between differentially expressed genes in HUVECs after transfection with s*iERG* or *siFLI1*. (D) Heatmap of Z-scores of common ERG and FLI1 target genes that are significantly differentially expressed in HUVECs after transfection with *siERG* or *siFLI1* relative to *siCTRL*. (E) Distribution of enriched GO biological process terms associated with differentially expressed genes following ERG (top) or FLI1 (bottom) knockdown in HUVECs. (F) Overlap between differentially expressed genes (identified using htseq-count (FDR<0.05 and FC>2) and differentially spliced genes (DSG, *i.e.* genes with at least one ERG-regulated ASE) identified by rMATS (with at least 15 reads supporting the event, FDR<0.05 and |ΔPSI|>10%) in HUVECs following *ERG* or *FLI1* knockdown.

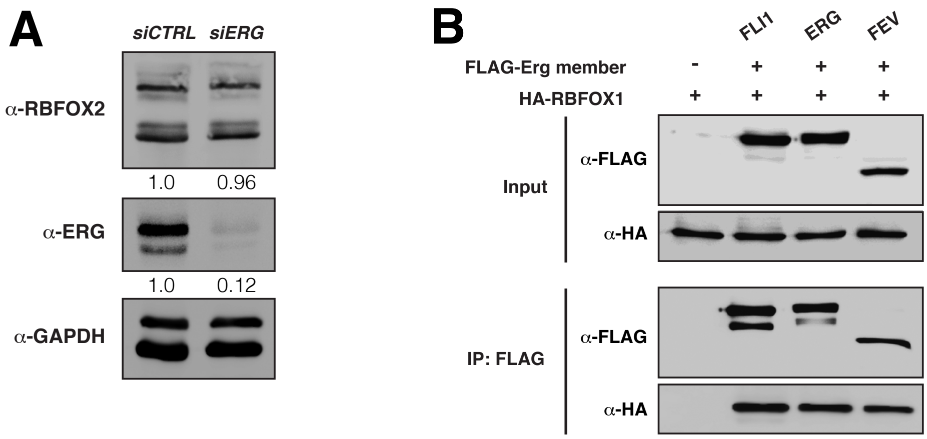

Supplementary Figure S5

Supplementary Figure S5: (A) Western blotting of RBFOX2, ERG and GAPDH, as control. Samples are total lysates from HeLa cells transfected with control (*siCTRL*) or specific ERG siRNA (*siERG*). Quantification of the RBFOX2/GAPDH and ERG/GAPDH ratios was performed by signal densitometry relatively to the *siCTL* condition and is indicated. (B) Immunoprecipitation of FLAG-tagged FLI1, ERG and FEV and anti-Flag and anti-HA western blotting. Samples are lysates from HEK293 cells transfected with HA-RBFOX1 together with the FLAG empty vector or with FLAG-tagged FLI1, ERG or FEV.

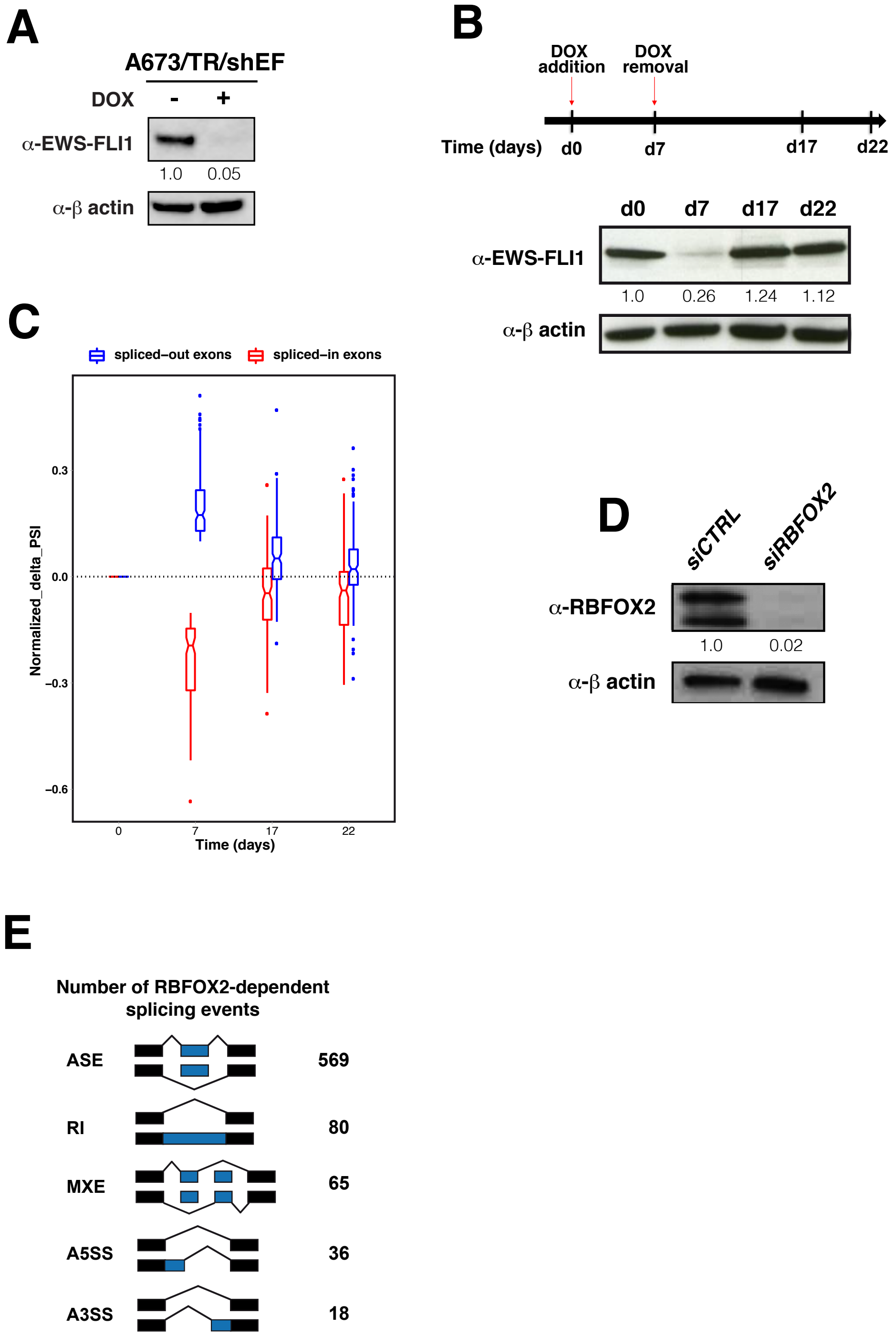

Supplementary Figure S6

Supplementary Figure S6: (A) Western blotting of EWS-FLI1 in A673/TR/shEF cells at day0 or day7 of doxycycline treatment. Actin was used as loading control. (B) Western blotting of EWS-FLI1 in A673/TR/shEF cells at day0 (-Dox) or day7 (+Dox) of doxycycline treatment. Actin was used as loading control. Quantification of the EWS-FLI1/β-actin ratios was performed by densitometry relatively to the day0 condition. (C) Effects of EWS-FLI1 re-expression on EWS-FLI1-dependent splicing events. Doxycycline has been removed at day7 and ΔPSI of EWS-FLI1-dependent splicing were analyzed after 10 days (day17) and 14 days (day22) after doxycycline removal. Alternatively spliced exons were recovered to their basal splicing pattern after EWS-FLI1 rescue expression (day17). Splicing analysis was performed with rMATS using day7 as reference data (EWS-FLI1-depleted cells). (D) Western blotting of RBFOX2 in A673/TR/shEF cells following control (*siCTRL*) or specific *RBFOX2* siRNA (*siRBFOX2*) transfection. Actin was used as loading control. Quantification of the RBFOX2/β-actin ratios was performed by signal densitometry relatively to the *siCTL* condition. (E) Numbers of significantly differentially spliced events identified after RBFOX2 inhibition in A673/TR/shEF cells. ASE: Alternatively spliced exons, RI: Retained intron, MXE: Mutually exclusive exons, A5SS: Alternative 5' splice site, A3SS: Alternative 3' splice site.

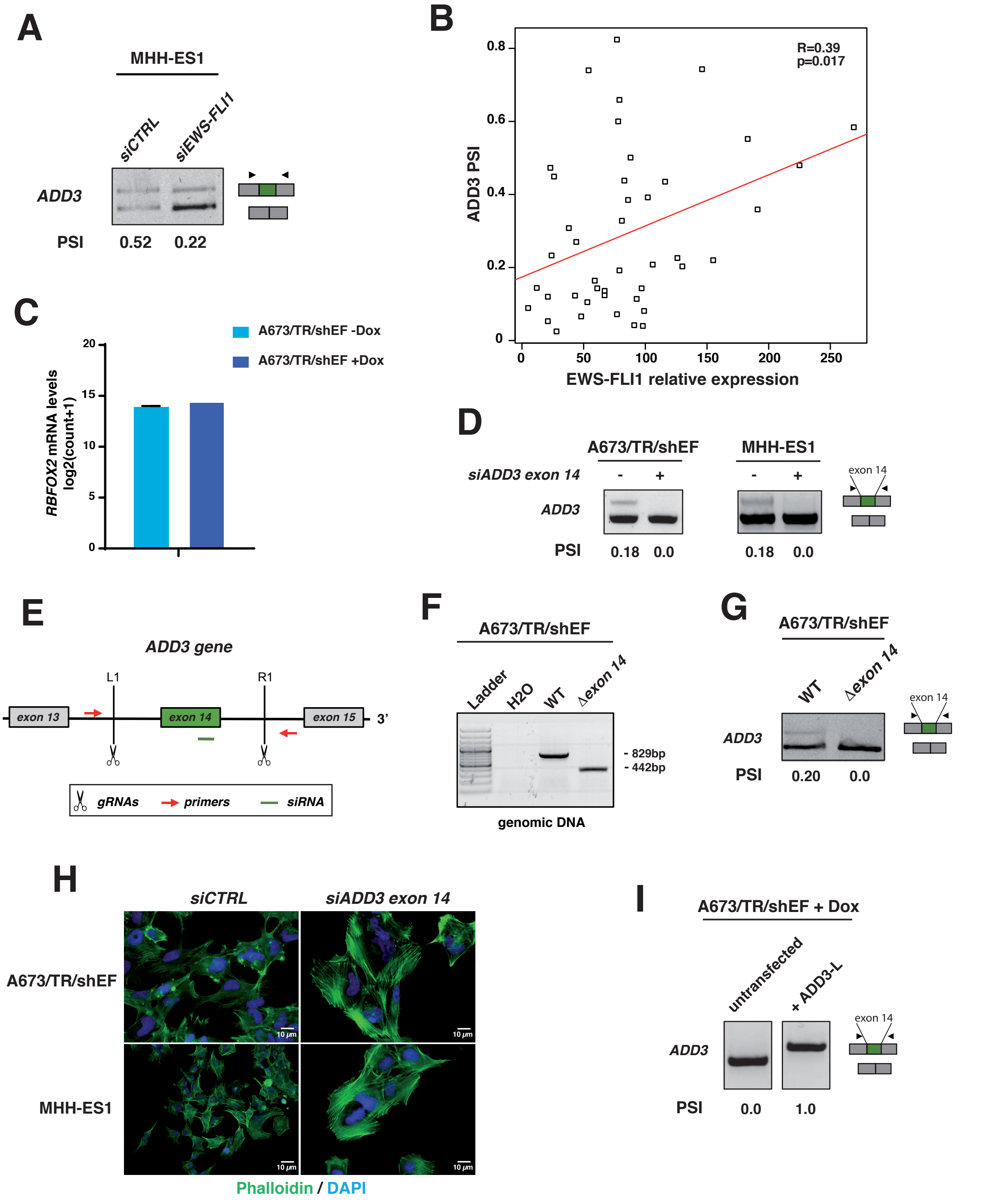

Supplementary Figure S7

Supplementary Figure S7: (A) RT-PCR analysis of *ADD3-L* splicing isoform in the MHH-ES1 Ewing sarcoma cell line after EWS-FLI1 depletion. Samples are RNA from MHH-ES1 cells transfected with a control *siRNA* or a specific *EWS-FLI1* siRNA. (B) Correlation between *ADD3* PSI values and EWS-FLI1 relative expression (number of junction reads supporting the fusion) in RNA-Seq data of Ewing sacoma patients. (C) Analysis of mRNA expression of *RBFOX2* in A673/TR/shEF cells non treated (A673/TR/shEF -DOX) or treated with doxycycline for 7 days (A673/TR/shEF +DOX) using RNA-seq analysis. (D) RT- PCR analysis of *ADD3-L* splicing. Samples are RNA from EWS-FLI1 expressing cells (A673/TR/shEF and MHH-ES1) transfected with control *siRNA* (-) or specific siRNA targeting exon 14 of *ADD3* (*siADD3 exon14*; +). (E) Design of CRISPR-Cas9 strategy to target *ADD3* exon 14. (F) PCR of *ADD3 exon 14* genomic region showing homozygous deletion in the A673/TR/shEF clone after using the CRISPR-Cas9 approach to delete *ADD3-exon 14* region. Samples are DNA extracts from A673/TR/shEF cells and positive clone for exon 14 of *ADD3* genomic region deletion (A673/TR/shEF Δ*exon 14 ADD3*). (G) RT- PCR analysis of *ADD3-L* splicing showing a complete loss of the *ADD-L* isoform in the A673/TR/shEF Δ*exon14 ADD3* clone. Samples are RNA from A673/TR/shEF cells and positive clone for exon 14 of *ADD3* genomic region deletion (A673/TR/shEF Δ*exon14 ADD3*). (H) Immunofluorescence of actin filaments stained with phalloidin (green channel) and DAPI (blue channel) of A673/TR/shEF and MHH-ES1 Ewing sarcoma cell lines treated with either control siRNA (*siCTRL*) or specific siRNA targeting ADD3-L isoform (*siADD3 exon14*). (I) RT- PCR analysis of *ADD3-L* splicing. Samples are RNA from Doxycycline-treated A673/TR/shEF untransfected or transfected with a ADD3-L-expression vector (+ ADD3-L).
